# Glycinergic Transmission in the Presence and Absence of Functional GlyT2: Lessons From the Auditory Brainstem

**DOI:** 10.3389/fnsyn.2020.560008

**Published:** 2021-02-09

**Authors:** Sina E. Brill, Ayse Maraslioglu, Catharina Kurz, Florian Kramer, Martin F. Fuhr, Abhyudai Singh, Eckhard Friauf

**Affiliations:** ^1^Animal Physiology Group, Department of Biology, University of Kaiserslautern, Kaiserslautern, Germany; ^2^Electrical & Computer Engineering, University of Delaware, Newark, DE, United States

**Keywords:** glycine transporter, glycinergic synapses, inhibitory synapses, lateral superior olive, neurotransmitter re-uptake, replenishment, short-term depression, fast synaptic transmission

## Abstract

Synaptic transmission is controlled by re-uptake systems that reduce transmitter concentrations in the synaptic cleft and recycle the transmitter into presynaptic terminals. The re-uptake systems are thought to ensure cytosolic concentrations in the terminals that are sufficient for reloading empty synaptic vesicles (SVs). Genetic deletion of glycine transporter 2 (GlyT2) results in severely disrupted inhibitory neurotransmission and ultimately to death. Here we investigated the role of GlyT2 at inhibitory glycinergic synapses in the mammalian auditory brainstem. These synapses are tuned for resilience, reliability, and precision, even during sustained high-frequency stimulation when endocytosis and refilling of SVs probably contribute substantially to efficient replenishment of the readily releasable pool (*RRP*). Such robust synapses are formed between MNTB and LSO neurons (medial nucleus of the trapezoid body, lateral superior olive). By means of patch-clamp recordings, we assessed the synaptic performance in controls, in GlyT2 knockout mice (KOs), and upon acute pharmacological GlyT2 blockade. Via computational modeling, we calculated the reoccupation rate of empty release sites and *RRP* replenishment kinetics during 60-s challenge and 60-s recovery periods. Control MNTB-LSO inputs maintained high fidelity neurotransmission at 50 Hz for 60 s and recovered very efficiently from synaptic depression. During 'marathon-experiments' (30,600 stimuli in 20 min), *RRP* replenishment accumulated to 1,260-fold. In contrast, KO inputs featured severe impairments. For example, the input number was reduced to ~1 (vs. ~4 in controls), implying massive functional degeneration of the MNTB-LSO microcircuit and a role of GlyT2 during synapse maturation. Surprisingly, neurotransmission did not collapse completely in KOs as inputs still replenished their small *RRP* 80-fold upon 50 Hz | 60 s challenge. However, they totally failed to do so for extended periods. Upon acute pharmacological GlyT2 inactivation, synaptic performance remained robust, in stark contrast to KOs. *RRP* replenishment was 865-fold in marathon-experiments, only ~1/3 lower than in controls. Collectively, our empirical and modeling results demonstrate that GlyT2 re-uptake activity is not the dominant factor in the SV recycling pathway that imparts indefatigability to MNTB-LSO synapses. We postulate that additional glycine sources, possibly the antiporter Asc-1, contribute to *RRP* replenishment at these high-fidelity brainstem synapses.

## Introduction

Inhibitory glycinergic neurotransmission is prominent in the mammalian brainstem, spinal cord, and some other regions. It plays a role in motor rhythm generation and sensory processing, for example in the pain pathway, the retina, and auditory nuclei involved in sound localization (Becker, 1990; Wässle et al., 1998; Zeilhofer et al., 2012; Vandenberg et al., 2014; Friauf et al., 2019). Glycinergic neurons release their transmitter molecules from presynaptic axon terminals through fast exocytosis of synaptic vesicles (SVs). This leads to a rapid increase of the glycine concentration in the synaptic cleft (~3 mM; Beato, 2008) and activation of strychnine-sensitive glycine receptors (GlyRs). Upon opening, GlyRs mediate Cl^−^ influx into the postsynaptic neuron and generate inhibitory postsynaptic currents (IPSCs). In essence, the postsynaptic cell is hyperpolarized and inhibited. Two glycine transporters (GlyT1 and GlyT2) act together in regulating synaptic and non-synaptic glycine concentrations. Both transporters belong to the large SLC6 family of Na^+^/Cl^−^-dependent transporters (Nelson, 1998) and perform distinct functions, as indicated by unique expression patterns (Zafra et al., 1995). GlyT1 is associated with glial cells and coupled to the co-transport of 2 Na^+^ ions and 1 Cl^−^ ion (Roux and Supplisson, 2000), thus reducing the extracellular glycine concentration to ~100 nM (vandenberg et al., 2016). In contrast, GlyT2 is largely located at axon terminals and a reliable marker for glycinergic neurons (Poyatos et al., 1997; Mahendrasingam et al., 2000; Zeilhofer et al., 2005). Its transport activity is unilateral and coupled to the co-transport of 3 Na^+^ ions and 1 Cl^−^ ion, which allows it to reduce glycine concentrations in the synaptic cleft to 10 nM and to maintain 20-40 mM in axon terminals. GlyT2 knockout mice develop spasticity and tremor and die at the end of the second postnatal week (Gomeza et al., 2003b). Loss of GlyT2 reportedly results in impaired refilling of glycinergic SVs which leads to severely disrupted neurotransmission (Gomeza et al., 2006; Rousseau et al., 2008; Aubrey, 2016). It therefore appears that GlyT2 is crucial for ensuring glycine concentrations in the axon terminal cytosol that are sufficient for loading SVs with glycine via the vesicular inhibitory amino acid transporter VIAAT (Supplisson and Roux, 2002). Mutations in *SLC6A*, the GlyT2-encoding gene, define a presynaptic component of hyperekplexia (or startle disease) in humans (Rees et al., 2006; Carta et al., 2012) as well as congenital muscular dystonia type 2 in cattle (Harvey et al., 2008).

Localization of high-frequency sounds in space is achieved by computing interaural level differences. In the mammalian auditory brainstem, the process involves proper function of a prominent glycinergic input from the medial nucleus of the trapezoid body (MNTB) to the lateral superior olive (LSO; Fischer et al., 2019; Friauf et al., 2019). MNTB-LSO synapses are tuned for resilience, reliability, and precision (Lujan and von Gersdorff, 2017) and have become a model system for analyzing glycinergic neurotransmission in general. During sustained high-frequency stimulation (50–200 Hz for 60 s), they are resistant to synaptic fatigue and perform remarkably faithfully (Kramer et al., 2014). High synaptic fidelity and exquisite temporal acuity are achieved via presynaptic mechanisms, namely a high number of SVs released by an action potential and rapid replenishment of the SV pool. These features appear to be unmatched by other synapse types (Krächan et al., 2017; Brill et al., 2019). What makes the MNTB-LSO inputs resistant to synaptic fatigue under high-frequency challenge is unclear. The high fidelity may be achieved by a specific mode of SV recycling, and GlyT2 may be one of the molecular key players. Indeed, GlyT2 is heavily abundant at MNTB axon terminals (Friauf et al., 1999).

In the present study, we assessed the role of GlyT2 for the performance of MNTB-LSO inputs during sustained high-frequency activation. We hypothesized that loss of GlyT2 activity would interfere with glycine recycling and heavily impair robust glycinergic neurotransmission at MNTB-LSO inputs, particularly under high-frequency challenge. We reasoned that postsynaptic responses should depress most drastically when stimulation occurred over time scales during which SV endocytosis and refilling with neurotransmitters are important (Murthy and Stevens, 1998; Edwards, 2007; Liang et al., 2017; Orlando et al., 2019). To address the issue, we performed whole-cell patch-clamp recordings from mouse LSO principal neurons in acute brain slices at postnatal day 11 and stimulated MNTB fibers electrically. We compared between normal mice (Ctrls), GlyT2-knockout mice (KOs) and mice in which GlyT2 activity was blocked pharmacologically. First, we assessed basic synaptic properties like quantal size and IPSC decay time. Then, we challenged the inputs in 60-s trains at 50 Hz and subsequently offered 60-s recovery periods during which test stimuli were applied at 1 Hz. In ‘marathon' experiments (Kramer et al., 2014), 10 such challenge/recovery trains were presented in a row, amounting to 30,600 stimuli within 20 min. By means of computational modeling, we determined the reoccupation rate of vacant release sites and the replenishment of the readily releasable pool (*RRP*). As expected, KOs displayed drastically impaired neurotransmission compared to Ctrls. The latter managed to maintain high fidelity transmission. Nevertheless, KOs were still able to replenish the *RRP* manifold. Unexpectedly, synaptic performance upon acute pharmacological inactivation of GlyT2 remained robust, even during marathon-experiments, in which *RRP* replenishment amounted to ~2/3 of the Ctrl value. The findings imply glycine sources other than GlyT2 which enable efficient refilling of the *RRP*.

## Materials and Methods

### Animals

Animal breeding and experiments were approved by the regional council according to the German animal protection act (TSchG § 4, Absatz 3) and in accordance with EU Directive 2010/63/EU. Animals were raised in the animal facilities of the University of Kaiserslautern and housed on a 12 h light-dark cycle with *ad libitum* access to food and water. Experiments were performed on C57BL/6N and Sv129/OLA mice of both sexes at postnatal day 11 ± 1. In the latter strain, the exon encoding the fourth transmembrane region was replaced with a PGK-neomycin resistance gene (Gomeza et al., 2003a). Homozygous KO mice lack the functional GlyT2 isoform. For immunohistochemistry, homozygous littermates (GlyT2^+/+^) formed the Ctrl group. In electrophysiological experiments, either homozygous littermates (GlyT2^+/+^) or wildtype C57BL/6N mice were used as Ctrls. During data acquisition and analysis, the investigator was blind to the genotype (except for experiments with pentobarbital).

### Immunohistochemistry

Deeply anesthetized mice (7% chloral hydrate, 0.01 ml/g body weight, intraperitoneal) were transcardially perfused with 150 mM phosphate-buffered saline (PBS, pH 7.4, room temperature), followed by ice-cold 4% paraformaldehyde (PFA) for 20 min (Ecoline VC-360 pump, IsmaTec, Chicago, USA). Brains were removed from the skull, postfixed for 2 h in 4% PFA, and stored overnight in 30% sucrose-PBS. Coronal brainstem slices were cut at 40 μm with a sliding microtome (HM 430, Thermo Fisher Scientific, Carlsbad, CA, USA) and transferred into 15% sucrose-PBS for 5 min; 3 × 10 min rinse steps in PBS followed at room temperature. Antibodies against GlyT2 (1:200, host: mouse, Synaptic Systems, Göttingen, Germany) and VIAAT (1:2,000, host: rabbit, Synaptic Systems) were applied free-floating at 4°C overnight in blocking solution (0.3% Triton-X-100, 5% goat serum, 1% bovine serum albumin (BSA) in PBS), followed by rinsing 3 × 10 min in PBS at room temperature. Slices were then incubated in the dark for 2 h in blocking solution and secondary antibody (1:1,000; goat-anti-mouse, Alexa Fluor 488; goat-anti-rabbit, Alexa Fluor 647, Thermo Fisher Scientific) and rinsed 3 × 10 min in PBS. Slices were then mounted on gelatin-coated glass slides and covered with mounting medium containing 2.5% DABCO (1,4-diazabicyclo[2.2.2]octane; Sigma-Aldrich, St. Louis, MO, USA). Images were acquired with a Zeiss LSM700 confocal microscope equipped with an EC Plan-Neofluar 40 × /1.3 Oil objective (Carl Zeiss Microscopy, Jena, Germany).

### Electrophysiology

Whole-cell patch-clamp recordings were performed from LSO principal neurons in acute brain slices near physiological temperature (36 ± 1°C). Coronal slices containing MNTB and LSO were prepared and processed as described previously (Hirtz et al., 2011). They were stored at room temperature in carbogenated artificial cerebrospinal fluid (ACSF) containing (in mM) 125 NaCl, 2.5 KCl, 1.25 NaH_2_PO_4_, 2 sodium pyruvate, 3 myo–inositol, 0.44 L (+) ascorbic acid, 25 NaHCO_3_, 10 glucose (H_2_O), 1 MgCl_2_, 2 CaCl_2_ (pH = 7.4). For recordings, individual slices were transferred into the recording chamber superfused with carbogenated ACSF. The recording chamber was mounted on an upright microscope (Eclipse E600FN, Nikon, Tokyo, Japan or Axioscope 2 FS, Zeiss) equipped with IR-DIC contrast optics (Nikon 4x CFI Achromat, 0.1 ∞; 60x CFI Fluor W, 1.00W ∞; Zeiss Fluar CFI 5x/0.25 ∞/0.17; Olympus LUMPlanFL N 60x/1.00W ∞/0/FN26.5). Slices were visualized with a CCD camera (C5405-01 or Orca-05G, Hamamatsu, Hamamatsu City, Japan).

LSO principal neurons were identified by their spindle-shaped somata and biophysical properties (Sterenborg et al., 2010). Stimulation pipettes (theta-glass 1.5 mm 6IN, WPI, Sarasota, FL, USA or GB150(F)-8P, Science Products, Hofheim am Taunus, Germany) and patch pipettes (GB150(F)-8P, Science Products) were pulled with a P-87 horizontal puller (Sutter Instruments Co., Novato, CA, USA). The tip diameter of the stimulation pipettes was ~ 20 μm. Patch pipette resistances ranged from 3 to 7 MΩ when filled with internal solution containing (in mM) 140 K-Gluconate, 10 HEPES, 5 EGTA, 1 MgCl_2_, 2 Na_2_ATP, 0.3 Na_2_GTP (280 ± 10 mOsm/L). The above solution (2 mM [Cl^−^]_i_) was used for most recordings (Figures 3, 4, 6–10). In a subset of experiments (Figures 2, 3D–F), the Cl^−^ driving force was increased by changing [Cl^−^]_i_ to 132 mM with a pipette solution of (in mM) 130 KCl, 10 HEPES, 5 EGTA, 1 MgCl_2_, 2 Na_2_ATP, 0.3 Na_2_GTP (280 ± 10 mOsm/L). With virtually symmetric Cl^−^ concentrations in the extracellular and pipette solution, both inhibitory and excitatory inputs generate inward currents at a holding potential of −70 mV, making it unable to distinguish between them. We therefore blocked AMPA/kainate glutamate receptors with CNQX (10 μM). Liquid junction potentials (15.4 mV for 2 mM, −3.5 mV for 132 mM Cl^−^ internal solution) were corrected offline (Axopatch-1D amplifier, Molecular Devices, San Jose, CA, USA) or online (EPC10 amplifier, HEKA Elektronik, Lambrecht/Pfalz, Germany). If not stated otherwise, chemicals were obtained from AppliChem (Darmstadt, Germany). Data were recorded with an Axopatch-1D or an EPC10 amplifier and visualized with ClampEX (Molecular Devices) or PatchMaster software (HEKA Elektronik). Recordings were sampled at 10–40 kHz and low-pass filtered at 2.9-7.2 kHz. Voltage-clamp recordings were performed at a holding potential of−70 mV. Series resistances amounted up to 25 MΩ and were compensated >30%.

Recordings were performed in voltage-clamp mode. Stimulation pipettes filled with ACSF were positioned at the lateral edge of the MNTB to evoke inhibitory postsynaptic currents (eIPSCs; Kramer et al., 2014). Stimuli consisted of 100-μs biphasic current pulses applied through a programmable pulse generator (Master 8, A.M.P.I, Jerusalem, Israel) connected to a stimulus isolator unit (A360, WPI). In order to determine the single fiber strength of MNTB-LSO inputs, monophasic current pulses were applied at 0.5 Hz and current amplitudes were increased in small increments (STG4004, Multichannel Systems, Reutlingen, Germany). Between 5 and 300 μA, the amplitude was increased in 5 μA increments and each amplitude was offered ten times. Above 300 μA, increments were 10 μA and five stimuli were applied. Prior to determining the performance of MNTB-LSO inputs during sustained high-frequency stimulation, stimulus amplitudes were adjusted during low-frequency stimulation to achieve stable and robust eIPSC amplitudes (stimulus amplitudes: 0.1-10 mA). Therewith, responses were clearly suprathreshold but also submaximal (neither minimal nor maximal stimulation). During an initial stimulation period (40 s | 1 Hz, 60 s | 1 Hz or 60 s | 0.2 Hz), eIPSC amplitudes were averaged to a 100% baseline. Thereafter, synapses were stimulated for 60 s at 50 Hz (3,000 stim). This frequency is semi-natural and possibly biologically relevant because the spontaneous *in vivo* firing rate of postnatal day 11 MNTB neurons ranges from 0.08 to 107 s^−1^ with the median at 35-37 s^−1^ (Sonntag et al., 2009; see also Bach and Kandler, 2020). Each 60 s | 50 Hz challenge period was followed by a 60-s recovery period during which test stimuli were presented at 1 Hz (60 stim). In marathon-experiments, ten such challenge/recovery trains were offered back to back, thus involving 30,600 stim over 20 min. In pharmacological experiments, GlyT2 was blocked with ALX1393 (O-[(2-Benzyloxyphenyl-3-flurophenyl)methyl]-L-serine; Sigma Aldrich), which was applied to the bath 10 min before and throughout the recordings. ALX blocks GlyT2 with an IC_50_ of 10–100 nM and is ~200-fold more sensitive for GlyT2 than for GlyT1 (Caulfield et al., 2001; Mingorance-Le Meur et al., 2013; vandenberg et al., 2016; Fratev et al., 2019; Mostyn et al., 2019). The concentration of 2 μM in ACSF used in the present study is very similar to the 1–3 μM used in other slice studies (Jeong et al., 2010; Oyama et al., 2017). In another series of experiments, spontaneous IPSCs (sIPSCs) were recorded from KO LSO neurons with the GABA_A_ receptor (GABA_A_R) modulator pentobarbital (Pbt, 30 μM in ACSF; Fagron, Glinde, Germany).

### Data Analysis

sIPSCs recorded in 132 mM [Cl^−^]_i_ solution and in the absence of the sodium channel blocker tetrodotoxin were detected by MiniAnalysis 6.0.3 (Synaptosoft, Fort Lee, NJ, USA) under manual control, and further processed in Excel 2013 (Microsoft Corporation, Redmond, WA, USA) and Origin 2017G (OriginLab Corporation, Northampton, MA, USA). From such recordings, we determined sIPSC foot-to-peak amplitude, sIPSC τ, sIPSC rate, and quantal size *q*. sIPSC amplitude was determined for each individual event. sIPSC τ was analyzed when the decay phase could be fitted with an *R*^2^ > 0.85. The decay course was fitted to a double-exponential function from which we calculated the weighted decay time constant τ_w_ (Fischer et al., 2019). Fifteen to one hundred events per neuron went into the analysis. Single cell data represent the median of the sample. The sIPSC rate was determined as the reciprocal of the mean inter-event interval. *q* was determined from sIPSC amplitude distribution histograms through a Gaussian fit from 0 to the bin after the 1st local maximum (bin width: 5 pA; Krächan et al., 2017). We determined *q* from sIPSCs in the absence of tetrodotoxin, but discarded action potential-triggered events offline certainly by the Gaussian fits.

From eIPSCs recorded during baseline stimulation in 2 mM [Cl^−^]_i_ and detected by MiniAnalysis 6.0.3, we determined eIPSC τ. The analysis was performed as described above for sIPSCs. Foot-to-peak amplitudes of eIPSCs were automatically determined with a custom-written plugin (Dr. Alexander Fischer, Univ. Kaiserslautern) implemented in IGOR Pro 6.34A (WaveMetrics Inc., Lake Oswego, OR, USA) and normalized to the baseline (arithmetic mean = 100%). In the eIPSC peak amplitude analysis, all events were considered. In the fidelity analysis, however, eIPSC peak amplitudes had to be ≥2-fold noise level to be suprathreshold. Otherwise, the response was counted as a synaptic failure (fidelity = 0; 100% fidelity if no failure occurred). The noise level was measured as 95% of maximal bidirectional deflection of the baseline. For statistics, amplitudes and fidelity levels were determined as the arithmetic mean of values from the last 10 eIPSCs of challenge or recovery periods. The time course of recovery was fitted to a monoexponential function using Origin 2017G. Recovery behavior was assessed by four means (cf. Table 4 and Brill et al., 2019): (*a*) *RecovA*: last ten eIPSC recovery vs. baseline (= 100%); (*b*) *RecovB*: last 10 eIPSC recovery vs. last 10 eIPSC of preceding challenge; (*c*) *RecovC*: last 10 eIPSC recovery vs. eIPSC_1_ of preceding challenge; (*d*) Fractional recovery *FR* (Kushmerick et al., 2006) calculated as

$$\begin{array}{l} FR=\frac{last\;10\;eIPSCs\;recovery-last\;10\;eIPSCs\;challenge}{baseline-last\;10\;eIPSCs\;challenge}. \end{array}$$

The quantal content *m*, i.e., the number of SVs released per stimulus, was calculated as *m* = $\frac{eIPSC}{q}$. In order to address synaptic release properties quantitatively, we determined the readily releasable pool (*RRP)* and the release probability (*P*_*v*_*)*. The cumulative current induced upon release of the entire *RRP* (*I*_*RRP*_) was assessed via the Elmqvist and Quastel (1965). In brief, *I*_*RRP*_ was determined by forward extrapolation of a linear fit through eIPSC_1−2_, and $P_{\text{v}}=\frac{I_{RRP}}{ePSC_{1}}$. The number of SVs in the *RRP* (*N*_*RRP*_) calculates to $N_{RRP}=\;\frac{I_{RRP}}{q}$.

To determine the number of inputs, the single fiber strength, and the maximal amplitude of MNTB-LSO inputs, eIPSCs from experiments with stepwise increasing stimulus amplitude were analyzed, followed by a K-means based cluster analysis performed in Matlab R2018 (MathWorks, Natick, MA, USA) with a custom-written routine (Dr. Dennis Weingarten, Univ. Kaiserslautern; Müller et al., 2019). To do so, we assigned centroids to the dataset and applied an iterative optimization algorithm to minimize the error between centroids and data points. Cluster numbers between one and nine were tested, and those clusters at which the errors flattened as a function of cluster number (elbow criterion) were further investigated using silhouette plots. We used silhouette plots as a graphical aid to estimate the most likely cluster number per eye.

### Computational Modeling

In order to determine the replenishment rate of the three cohorts of synaptic inputs (Ctrls, KOs, ALX), we used SV-based computational modeling. A few experiments were performed in 132 mM [Cl^−^]_i_ (Figures 2, 3D–F), yet the majority in 2 mM [Cl^−^]_i_ (Figures 3, 4, 6, 10). To enable direct comparisons, we adjusted the *q* values. We did so by converting the sample mean *q* values for Ctrls and KOs obtained in 132 mM [Cl^−^]_i_ during the normalization period via multiplying with 0.6:

$$\frac{V_{hold\;}-\;E_{Cl(2\;mM\;{\left[Cl^{-}\right]}_{i})}}{V_{hold\;}-\;E_{Cl(132\;mM\;{\left[Cl^{-}\right]}_{i})}\;}=\;\frac{-70\;mV-(-112.2\;mV)}{-70\;mV-(-0.3\;mV)}\;=-0.6$$

We thus obtained *q* values of 16.5 pA and 17.1 pA, respectively (Table 3). For the ALX sample, *q* (14.0 pA) was directly determined from sIPSC amplitude distribution histograms obtained in 2 mM [Cl^−^]_i_ (Table 3).

Our model considers *M* release sites, where each site is occupied by an SV or empty. Each empty site is replenished at a rate *RR*_*i*_ between the *i*^*th*^ and (*i*+*1)*^*th*^ stimulus. *N*_*RRPi*_ denotes the number of occupied sites just before the arrival of the *i*^*th*^ action potential, and *P*_*v*_ is the probability of SV fusion upon an action potential. We assume that all release sites are occupied at the start of challenging, i.e., there is no empty site (*M* = *N*_*RRP*__1_). The latter assumption deviates from the docking site paradigm discussed previously (Pulido and Marty, 2018) which stipulates that some docking sites may be free at rest so that the number of docking sites does not need to match the RRP size.

Further,

(1) $$\begin{array}{l} N_{RRPi}*P_{v\;}=m_{i} \end{array}$$

(2) $$\begin{array}{l} N_{RRP_{i+1}}=N_{RRP_{i}}*\left(1-P_{v}\right)+\left(M-N_{RRP_{i}}*\left(1-P_{v}\right)\right)*\left(1-e^{-\;\frac{RR_{i}}{f}}\right) \end{array}$$

where *m*_*i*_ is the number of SVs released in response to the *i*^*th*^ stimulus (= quantal content) and *f* is the stimulus frequency (50 Hz).

The term *N*_*RRPi*+1_ = *N*_RRPi_*(1 − *P*_*v*_) represents *N*_*RRP*_ depletion in response to the i^th^ stimulus, and the term $\left(M-N_{RRP_{i}}*\left(1-P_{v}\right)\right)*\left(1-e^{-\;\frac{RR_{i}}{f}}\right)$ represents the net replenishment rate between the *i*^*th*^ and the *i*+*1*^*th*^ stimulus, with $M-N_{RRPi}*\left(1-P_{v}\right)=M-N_{RRPi}+\;N_{RRPi}^{*}P_{v}$ depicting the number of empty sites directly after SV release to the i^th^ stimulus and ${\left(M-N_{RRPi}+\;N_{RRPi}^{*}P_{v}\right)}^{*}\left(-e^{-\;\frac{RR_{i}}{f}}\right)$ depicting the decrease of empty sites through replenishment.

We assume that *RR* builds up initially (Weingarten, 2018) such that *RR*_1_, *RR*_2_, and *RR*_3_ differ. To obtain basic parameters and starting with the 4th stimulus, we assume that *RR* remains constant during the 1st second, namely *RR*_*i*_ = *RR*_4_. The model parameters *M, P*_*v*_*, RR*_1_, *RR*_2_, *RR*_3_, and *RR*_4_ are estimated by performing a least-square fitting with the averaged empirical data for the 1st second. To predict long-term SV release, we assume that *RR* decreases monotonically as per a double exponential decay via

(3) $$\begin{array}{l} RR_{i}=RR_{\min}+\left(RR_{4}-RR_{\min}\right)*\left(g*e^{-\;\frac{i-Delay}{\tau_{1}}}+\left(1-g\right)*e^{-\;\frac{i-Delay}{\tau_{2}}}\right) \end{array}$$

if stimulus *i* > *Delay*. τ_1_ and τ_2_ are decay time constants for *RR, g* is a constant, and *RR*_*min*_ is the minimum replenishment rate after a very large number of stimuli (going to infinity). If *i* < *Delay, RR* is assumed to be constant and equal to *RR*_4_. τ_1_, τ_2_, *g, Delay*, and *RR*_*min*_ are determined by fitting the model to the number of SVs released for the entire challenge period (all 3,000 stimuli).

To model the long-term dynamics of synaptic transmission, one must consider different upstream SV pools that feed into the RRP. Instead of explicitly modeling these upstream pools, we have taken a phenomenological approach here in which – after a certain delay – RR begins to decrease as per a double exponential (given by Equation 3). Biologically, this delay corresponds to the depletion of the upstream SV pool upon high-frequency stimulation, which subsequently causes RR to decrease. The double-exponential form of RR is indicative of two different upstream pools, with the kinetics of their refilling being captured by the two time constants τ_1_ and τ_2_.

To model replenishment during recovery periods, we again use Equations 1 and 2, but with *f* = *1 Hz*. *RR* is assumed to increase monotonically during each recovery period as per the following equation:

(4) $$\begin{array}{l} RR_{i}=\;RR_{recmax}+\left(RR_{recmin}-RR_{recmax}\right)*e^{-\;\frac{i-3,000}{\tau_{rec}}};\\ \;3,001\;\leq \;i\;\leq \;3,060 \end{array}$$

*RR*_*recmax*_*, RR*_*recmin*_, and τ_rec_ are estimated by fitting the model to the empirically obtained recovery data.

### Laser Microdissection and RNA Sequencing

MNTB tissue from postnatal day 11 ± 1 mice (*n* = 3) was collected via laser microdissection (LMD6500/DM6000B laser system; Leica Microsystems). RNA quality control and cDNA synthesis were done following routine protocols (Picelli et al., 2013). Library preparation, sequencing, and subsequent processing of sequencing data was done as described (Müller et al., 2019). Counts were normalized as reads per kilobase per million (RPKM) which allows to compare expression levels.

### Statistics

Statistical evaluation was performed if *n* ≥ 6 (Excel plug-in WinSTAT, R. Fitch Software). Outliers (> 4-times standard deviation above or below mean) were excluded from further analysis. Therefore, n numbers may vary in tables. The mean outlier rate was 1.2% (range 0-14.2%). Normally distributed data (Kolmogorov-Smirnov test) were compared using paired or unpaired 2-tailed *t*-tests. Otherwise, a Mann-Whitney U-test was applied for unpaired data. In case of multiple comparisons, critical α values were *post hoc* Šidák corrected (Abdi, 2007). Consequently, significance levels are as follows:

**Table:** 

**Significance level**	**Number of comparisons (k)**				
	**1**	**2**	**3**	**5**	**10**
*	0.05	0.0253	0.017	0.0102	0.0051
**	0.01	0.005	0.0033	0.002	0.001
***	0.001	5.0 × 10^−4^	3.3 × 10^−4^	2.0 × 10^−4^	1.0 × 10^−4^

Data smoothing was obtained through weighted moving averages over three (1 Hz) or nine (50 Hz) data points (Origin 2017G). Values for individual neurons are illustrated as open dots, sample data depict the arithmetic mean ± standard error of the mean (SEM) and are illustrated as diamonds with error bars. Numbers of events and recorded neurons (n) as well as significance levels are provided in the tables.

## Results

### Absence of GlyT2 Immunoreactivity in GlyT2 KO Mice

**Figure 1:**
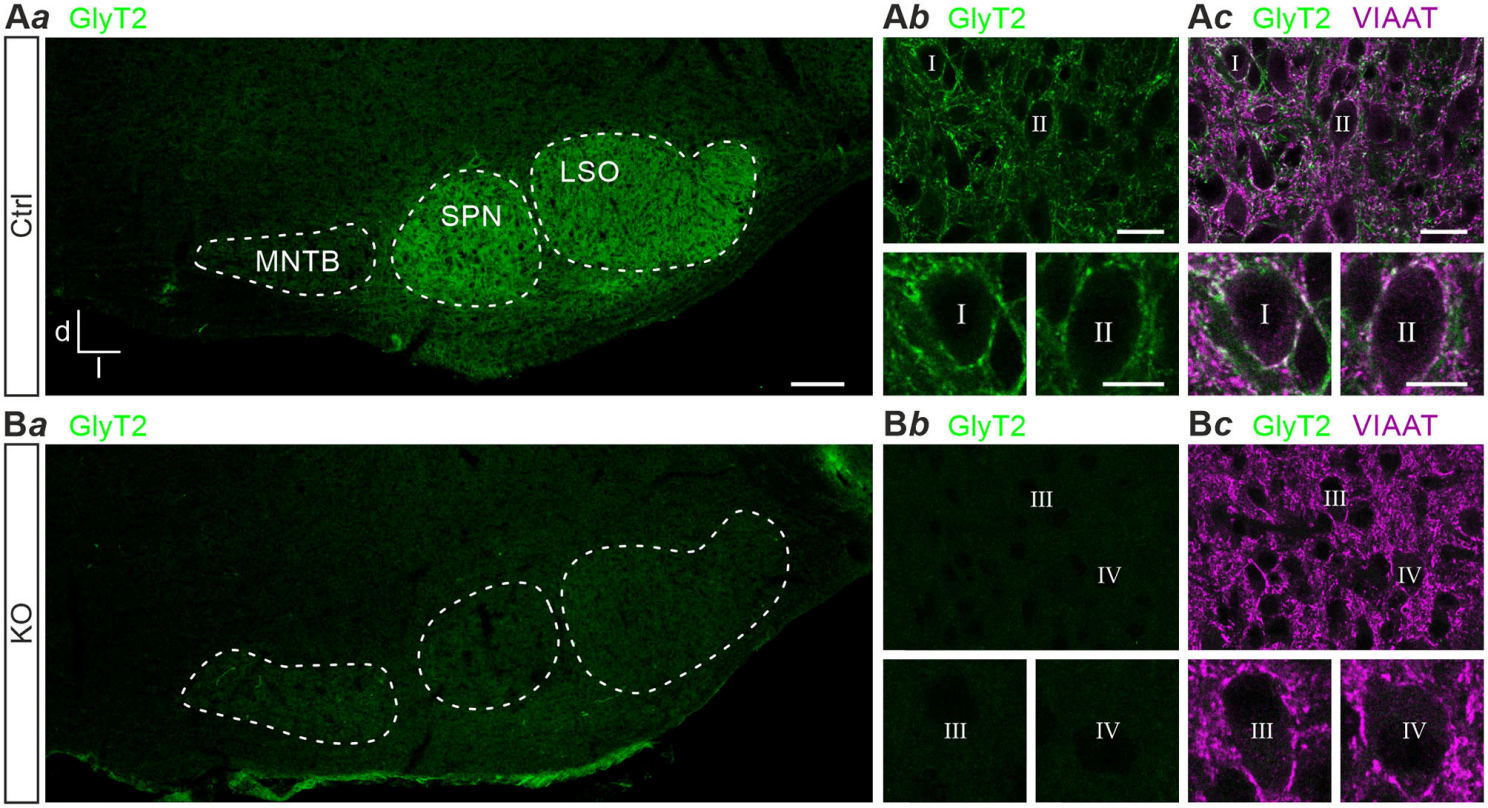
Immunohistochemistry in control and glycine transporter 2 (GlyT2) knockout mice. Immunofluorescence in auditory brainstem nuclei of control (Ctrl) and GlyT2 knockout (KO) mice **(A,B)**. GlyT2 immunoreactivity in the SOC **(A***a*,**B***a***)** and LSO **(A***b*,**B***b***)**, and double labeling for GlyT2 and VIAAT **(A***c*,**B***c***)**. Four representative LSO neurons (I–IV) at higher magnification. Scale bars: 100 μm **(A***a*,**B***a***)**, 20 μm **(A***b*,*c*,**B***b,c***)**, 10 μm (insets). Abbreviations: d, dorsal; l, lateral; LSO, lateral superior olive; MNTB, medial nucleus of the trapezoid body; SOC, superior olivary complex; SPN, superior paraolivary nucleus; VIAAT, vesicular inhibitory amino acid transporter.

Previous results obtained with polyclonal antibodies showed abundant GlyT2 immunoreactivity at glycinergic MNTB axon terminals in rats (Friauf et al., 1999). Using a monoclonal primary antibody, we here confirm the labeling pattern in the superior olivary complex of mice (Figure 1A*a*). In addition, immunohistochemical labeling with this antibody in GlyT2 KO mice revealed a complete absence of GlyT2 signals from the LSO, the adjacent superior paraolivary nucleus (SPN), and the surrounding reticular formation (Figures 1B*a*,*b*). These findings are in slight contrast to immunolabeling reported earlier in and around the LSO of GlyT2 KO mice (cf. Figure 4C of Gomeza et al., 2003b). In Ctrls, GlyT2 encrusted the somata and proximal dendrites of LSO principal cells (Figures 1A*b,c*) and codistributed with immunoreactive puncta for VIAAT (aka VGAT; Wojcik et al., 2006). The labeling pattern of VIAAT appeared unchanged in GlyT2 KOs (Figures 1B*b,c*), indicating that SV loading with glycine and GABA does not become abolished upon GlyT2 gene deletion.

### GlyT2 Gene Deletion Results in Fewer Spontaneous Events With Prolonged Decay Time, but the Quantal Size *q* Remains Unchanged

**Figure 2:**
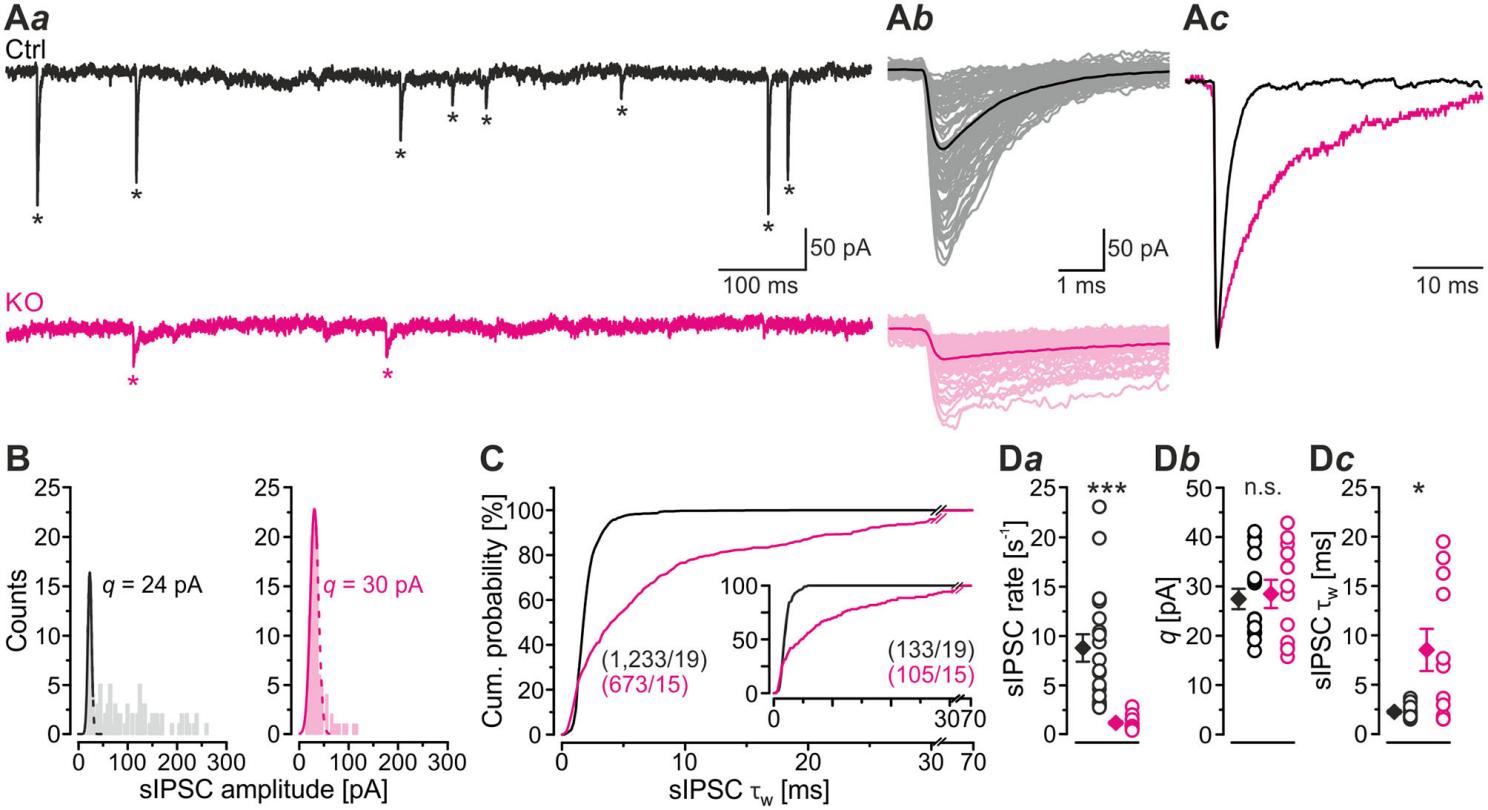
LSO neurons in GlyT2 KO mice display a lower rate and prolonged decay time of sIPSCs. **(A)** Current traces depicting spontaneous inhibitory postsynaptic currents (sIPSCs, asterisks) from a representative Ctrl (black) and KO (purple) neuron **(A***a***)**. Notice that traces were obtained in the absence of tetrodotoxin. Because recordings were done with 132 mM [Cl^−^]_i_, sIPSCs are inward directed. Overlay of individual sIPSCs (light traces) and graphical mean [dark traces, **(A***b***)**]. Peak-scaled images of graphical means **(A***c***)** to compare decay kinetics. **(B)** sIPSC peak amplitude distribution histograms for two representative neurons, from which the quantal size (*q*) was determined (see methods for details). **(C)** Cumulative probability of weighted decay time constant (τ_w_) based on unequal event numbers (Ctrl: 9–85 sIPSCs/neuron, KO: 7–100 sIPSCs/neuron) and seven events from each neuron (inset). Brackets: sIPSCs/neurons. **(D)** Sample data and statistics for rate **(D***a***)**, *q* **(D***b***)** as well as τ_w_ **(D***c***)** for Ctrl and KO samples. Details in Table 1. For meaning of *, ***, see Materials and Methods, Statistics.

In a first step of electrophysiological analysis, we determined basic synaptic properties of MNTB-LSO inputs in Ctrls and KOs. KO inputs displayed a drastically lower rate of spontaneous IPSCs (sIPSCs; ~8-fold; Figures 2A*a*,**D***a*; Table 1). KO inputs exhibited a narrow distribution of sIPSC peak amplitudes and reduced mean sIPSC peak amplitudes (~2-fold smaller; Figure 2A*b*; Table 1), probably due to loss of multivesicular release. However, the quantal size *q*, as determined from Gaussian diagrams, was statistically indistinguishable between both genotypes (Figures 2B,D*b*; Table 1). Similar to the immunohistochemical results, this again indicates that SV loading with glycine is not abolished upon GlyT2 gene deletion. The weighted decay time (τ_*w*_) of sIPSCs was considerably longer in KOs (~4-fold; Figures 2A*c*,**C,D***c*; Table 1), and the difference was due to a ~6-fold longer decay time constant τ_2_, whereas the short τ_1_ was unaltered (Table 1). The ratio τ_1_*/*τ_2_ was ~3-fold higher in Ctrls than in KOs (Table 1). The longer τ values contrast the results described in the original GlyT2 KO paper, which demonstrated unchanged decay kinetics for miniature IPSCs (mIPSCs; Gomeza et al., 2003b). Together, the above results emphasize several changes in inhibitory neurotransmission at MNTB-LSO synapses upon GlyT2 gene deletion, namely a lower spontaneous rate, smaller sIPSC amplitudes, and longer decay times. On the other hand, *q* remains unchanged. The cumulative decay time curves depicted in Figure 2C were obtained with different sIPSC numbers within the samples (Ctrl: 9-85 sIPSCs/neuron, KO: 7-100 sIPSCs/neuron). To account for a bias problem associated with such an unbalanced approach, we randomly picked seven sIPSCs for each neuron and replotted the curves (Figure 2C, inset). Also with this approach τ_*w*_ of sIPSCs was significantly longer in KOs (~4-fold; unpaired 2-tailed *t*-test; *P* = 0.002). Thus, the results confirmed the above findings obtained with unequal numbers.

**Table 1:** sIPSC properties of LSO principal neurons in Ctrl and KO mice.

		**Ctrl**	**KO**	**KO + Pbt**	**Ctrl vs. KO**	**KO vs. KO + Pbt**
					***P*** **value** ** significance**	
sIPSC rate [s^−1^]		8.7 ± 1.4 (1,800/18)	1.1 ± 0.2 (784/13)		4.1 × 10^−5^ ^***^	
sIPSC amplitude [pA]		90.8 ± 10.9 (1,800/18)	39.7 ± 4.2 (884/14)		2.4 × 10^−4^ ^***^	
*q* [pA]		27.5 ± 2.1	28.5 ± 2.9	27.7 ± 1.1	0.8 n.s.	0.8 n.s.
sIPSC τ [ms]	τ_w_	2.3 ± 0.2 (1,223/17)	8.5 ± 2.1 (754/11)	9.6 ± 2.8 (1,541/15)	0.01 ^*^	0.5 n.s.
	τ_1_ [%]	1.9 ± 0.1 [51 ± 1]	2.5 ± 0.4 [51 ± 1]	2.9 ± 0.6 [52 ± 1]	0.1 n.s.	0.5 n.s.
	τ_2_ [%]	3.6 ± 0.3 [49 ± 1]	20.7 ± 4.1 [49 ± 1]	23.5 ± 4.3 [48 ± 1]	0.002 ^**^	0.7 n.s.
Ratio τ_1_/τ_2_		0.54 ± 0.03	0.17 ± 0.04			

**Figure 3:**
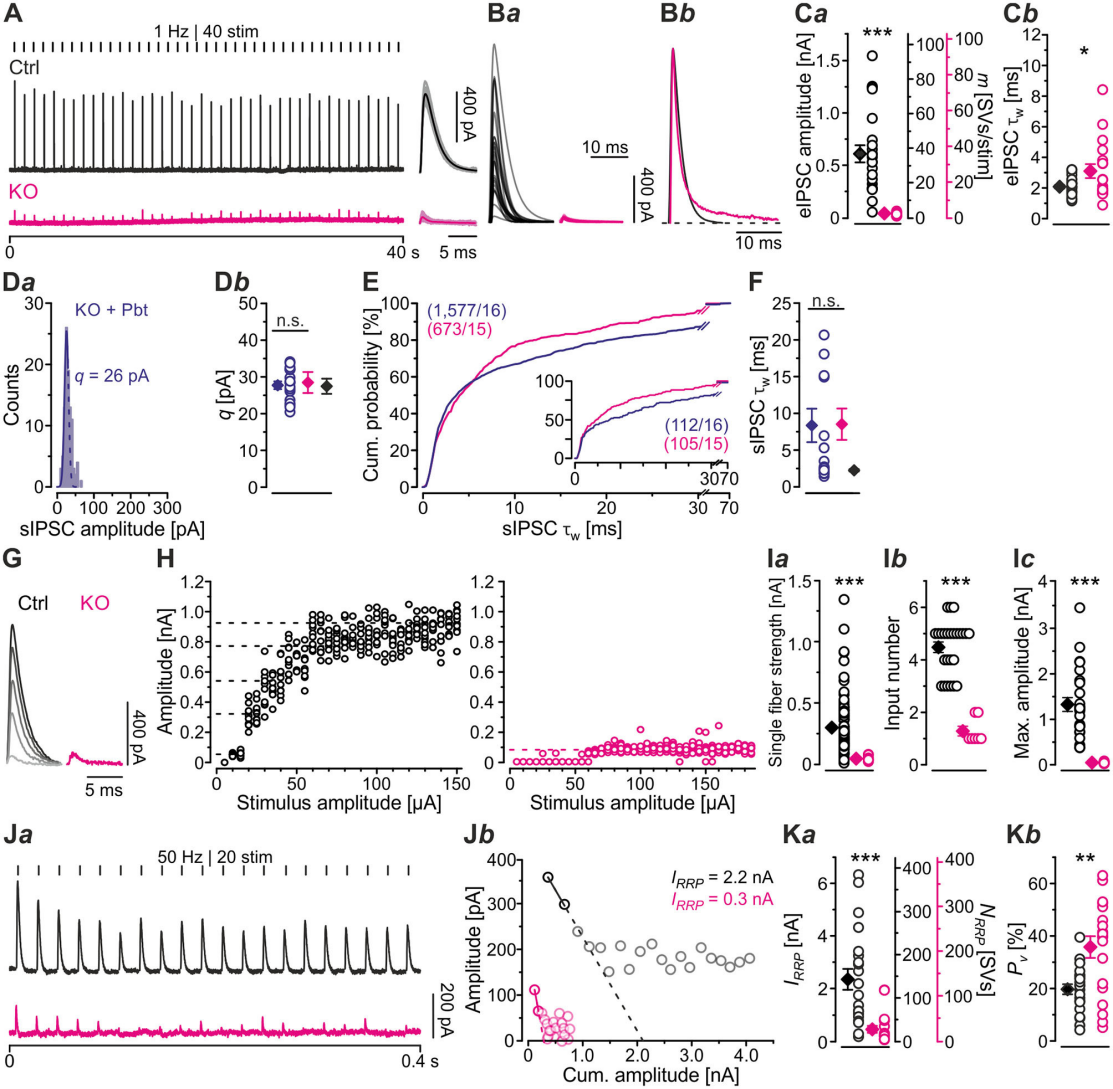
MNTB-LSO microcircuit and synaptic strength are severely impaired in KO mice. **(A)** Representative current traces from a Ctrl and a KO neuron, depicting evoked IPSCs (eIPSCs). Left parts show raw traces (1 Hz | 40 stim), right parts show individual eIPSCs and graphical means (light- and dark-shaded). In this [except panels **(D–F)**] and all subsequent figures, recordings were performed with 2 mM [Cl^−^]_i_; eIPSCs are therefore outward directed. Stimulus artifacts were blanked for clarity here and in subsequent figures. **(B)** Overlay of mean eIPSCs for the sample of Ctrl and KO neurons **(B***a***)** and peak-scaled eIPSCs (sample means), depicting kinetics for Ctrl and KO **(B***b***)**. Dashed line indicates resting level. **(C)** Sample data and statistics for eIPSC amplitude and quantal content [*m*, **(C***a***)**], as well as τ_w_ **(C***b***)**. Y-axes for *m* vary slightly due to small differences in *q* (see methods). **(D)** sIPSC peak amplitude distribution histograms for a representative KO neuron in the presence of the GABA_A_R modulator pentobarbital [30 μM Pbt, blue, **(D***a***)**] as well as sample data and statistics for *q* **(D***b***)**. Notice that recordings for panels **(D–F)** were done with 132 mM [Cl^−^]_i_ and were made in the absence of tetrodotoxin. **(E)** Cumulative probability of weighted decay time constant (τ_w_) based on unequal event numbers (KO: 7–100 sIPSCs/neuron, KO + Pbt: 16–145 sIPSCs/neuron) and seven events from each neuron (inset). Brackets: sIPSCs/neurons. **(F)** Sample data and statistics for τ_w_. **(G)** Current traces from a representative Ctrl and KO neuron, depicting eIPSCs elicited with gradually increasing stimulus amplitudes. Lighter shades correspond to low, darker shades to high stimulus amplitudes. **(H)** eIPSC amplitudes at gradually increasing stimulus amplitudes [same neurons as in **(G)**]. Dashed lines indicate distinct synaptic inputs. **(I)** Sample data and statistics for single fiber strength **(I***a***)**, input number **(I***b***)**, and maximal amplitude **(I***c***)**. **(J)** Estimation of readily releasable pool (*RRP*) size. Current traces from a representative Ctrl and KO neuron **(J***a***)**, depicting 20 eIPSCs during 50 Hz stimulation and corresponding Elmqvist-Quastel plots **(J***b***)**. The cumulative current amplitude (*I*_*RRP*_) upon complete *RRP* release was determined by linear regression of the first two eIPSCs. **(K)** Sample data and statistics for *I*_*RRP*_ and number of SVs in *RRP* [*N*_*RRP*_, **(K***a***)**], as well as release probability [*P*_*v*_, **(K***b***)**]. Y-axes for *N*_*RRP*_ vary slightly due to small differences in *q*. Details in Tables 1–3. For meaning of *, **, ***, see Materials and Methods, Statistics.

Mean values ± SEM of sIPSC rate, amplitude and ratio τ_1_/τ_2_ for Ctrl and KO samples, as well as quantal size q, sIPSC decay time constants τ_w_ (weighted time constant), τ_1_ and τ_2_ for Ctrl, KO, and KO in the presence of pentobarbital (KO + Pbt; cf. Figures 2, 3). Also depicted are P values and significance levels. Round brackets: number of events/neuron. Square brackets: amplitudes of τ_1_ and τ_2_ in %. Statistics: Ctrl vs. KO; KO vs. KO + Pbt: unpaired 2-tailed t-test. If applicable, Šidák correction [number of comparisons (k) = 2]. Recordings were done with 132 mM [Cl^−^]_i_ and in the absence of tetrodotoxin. For meaning of

*, **, ***, *see Materials and Methods, Statistics*.

### Inhibitory MNTB-LSO Inputs in GlyT2 KO Mice Are of Lower Amplitude and Display a Longer Decay Component

In a next series of experiments, we activated MNTB axons with maximal stimulus amplitudes at low frequency (1 Hz for 40–60 s) and determined peak amplitudes of evoked IPSCs (eIPSCs) in LSO principal cells. Peak amplitudes were drastically reduced in KOs (~16-fold, Figures 3A,B*a***,C***a*; Table 2). Moreover, KO eIPSCs had higher τ_*w*_ values (~1.5-fold, Figures 3B*b***,C***b*; Table 2), consistent with our findings for sIPSCs. The longer τ_*w*_ was due to an almost 4-fold longer slow decay time τ_2_, whereas the fast decay time τ_1_ was statistically indistinguishable (Table 2). As postsynaptic receptors with longer open times coexisted with those featuring short open times, two types of postsynaptic receptors appear to be present in GlyT2 KOs at postnatal day 11, whereas Ctrls harbor mainly receptors with short open times. We find it unlikely that a longer presence of glycine in the synaptic cleft of MNTB-LSO inputs alone causes the longer IPSCs. In GlyT1 KOs, weighted sIPSC decay time constants are 1.5-fold longer than in Ctrls and were explained by an increased accumulation of extracellular glycine, therewith leading to prolonged activation of GlyRs (Gomeza et al., 2003a). Our results show considerably prolonged τ_*w*_ values (~4-fold longer in KOs vs. Ctrls). We find it unlikely that presynaptic desynchronization attributes substantially to the eIPSC kinetics and favor another cause, namely receptor effects. As we will outline below, we find 1.3-fold prolonged τ_*w*_ values, similar to the ones in the Gomeza paper, upon acute pharmacological blockade of GlyT2 (cf. Figures 7D*b;* Table 3). Therefore, we reason that the drastically longer KO decay times for both sIPSCs and eIPSCs more likely point toward an altered molecular nature of postsynaptic receptors in the LSO, rather than merely a longer presence of the transmitter molecules in the synaptic cleft [NB: Gomeza et al. (2003b) reported unchanged sIPSC decay times in the hypoglossal nucleus of GlyT2 KO mice].

**Table 2:** Synaptic strength and release properties of MNTB-LSO inputs in Ctrl and KO mice.

		**Ctrl**	**KO**	***P* value significance**
eIPSC amplitude [pA]		609 ± 82 (23)	39 ± 3 (18)	5.4 × 10^−7^ ^***^
*m* [SVs/stim]		37 ± 5 (23)	2 ± 0 (18)	5.2 × 10^−7^ ^***^
eIPSC τ [ms]	τ_w_	2.1 ± 0.1 (23)	3.1 ± 0.5 (18)	0.04 ^*^
	τ_1_ [%]	1.9 ± 0.1 [48 ± 3]	1.7 ± 0.2 [71 ± 4]	0.4 n.s.
	τ_2_ [%]	2.2 ± 0.2 [52 ± 3]	8.5 ± 1.5 [29 ± 4]	6.4 × 10^−4^ ^***^
Single fiber strength [pA]		282 ± 27 (113/25)	54 ± 8 (9/7)	1.1 × 10^−8^ ^***^
Input number		4.5 ± 0.2 (25)	1.3 ± 0.2 (7)	5.2 × 10^−9^ ^***^
Maximal amplitude [pA]		1,330 ± 150 (25)	50 ± 8 (7)	1.3 × 10^−8^ ^***^

Mean values ± SEM of eIPSC amplitude, quantal content m, eIPSC τ, single fiber strength, input number, and maximal eIPSC amplitude for Ctrl and KO samples (cf. Figure 3). Also depicted are P values and significance levels. eIPSC parameters were determined during low-frequency baseline stimulation with maximal stimulus amplitude (0.2 Hz or 1 Hz). Round brackets: number of fibers/neuron or number of neurons. Square brackets: amplitudes of τ_1_ and τ_2_ in %. Statistics: Ctrl vs. KO: unpaired 2-tailed t-test. Recordings were done with 2 mM [Cl^−^]_i_. For meaning of

*, ***, *see Materials and Methods, Statistics*.

**Table 3:** Synaptic strength and release properties of MNTB-LSO inputs in Ctrl, KO, and ALX.

		**Ctrl**	**KO**	**ALX**	**Ctrl vs. KO**	**Ctrl vs. ALX**
					***P*** **value** **significance**	
*I_*RRP*_* [nA]		2.4 ± 0.1 (21)	0.5 ± 0.0 (19)	2.4 ± 0.1 (11)	1.3 × 10^−4^ ^***^	0.9 n.s.
*N_*RRP*_* [SVs]		144 ± 5 (21)	27 ± 2 (19)	172 ± 7 (11)	1.1 × 10^−4^ ^***^	0.5 n.s.
*P_*V*_* [%]		19.7 ± 0.4 (21)	35.8 ± 0.9 (19)	13.7 ± 0.5 (11)	0.002 ^**^	0.05 n.s.
*q* [pA]		16.5^#^	17.1^#^	14.0		
eIPSC amplitude [pA]		279 ± 41 (18)		222 ± 38 (15)		0.3 n.s.
*m* [SVs/stim]		17 ± 3 (18)		16 ± 3 (15)		0.8 n.s.
eIPSC τ [ms]	τ_w_	1.8 ± 0.1 (17)		2.3 ± 0.1 (15)		0.01 ^*^
	τ_1_ [%]	1.8 ± 0.1 [49 ± 4]		2.2 ± 0.1 [48 ± 5%]		0.06 n.s.
	τ_2_ [%]	1.8 ± 0.1 [51 ± 4]		2.4 ± 0.2 [52 ± 5%]		0.008 ^**^
**Docking site model**						
		**Ctrl**	**KO**	**ALX**		
*N_*RRP*1_* [SVs]		139	32	155		
*m_1_* [SVs/stim]		20	7	18		
*P_*V*_* [%]		14	22	12		

Mean values ± SEM of cumulative current amplitude (I_RRP_) upon complete release of the RRP, number of SVs in RRP (N_RRP_), and release probability (P_v_) for Ctrl, KO, and ALX samples (cf. Figures 3, 7), including P values and significance levels. eIPSC amplitude, m, eIPSC τ for Ctrl and ALX samples (cf. Figure 7). eIPSC parameters were determined during low-frequency baseline stimulation (0.2 or 1 Hz). Round brackets: number of neurons. Square brackets: amplitudes of τ_1_ and τ_2_ in %. Statistics: Ctrl vs. KO, Ctrl vs. ALX: unpaired 2-tailed t-test. If applicable, Šidák correction (k = 2). Recordings were done with 2 mM [Cl^−^]_i_ and from different cohorts than in Table 2. ^#^Sample mean q values obtained from 132 mM [Cl^−^]_i_ recordings in the absence of tetrodotoxin were mathematically converted to match the situation in 2 mM [Cl^−^]_i_ (cf. Table 1 and Methods). Synaptic stimulation was done at half-maximal stimulus amplitudes. For comparison, some relevant numbers obtained via a docking site model are also shown. For meaning of

*, **, ***, *see Materials and Methods, Statistics*.

GABA_A_Rs would explain longer IPSCs, yet GlyRs composed of ‘fetal' α_2_ subunits – instead of ‘adult' α_1_ subunits – would also do so (Becker et al., 1988; Takahashi et al., 1992). Recently, we found no evidence for synaptic GABA_A_R-mediated signaling at MNTB-LSO inputs of normal mice (Fischer et al., 2019; for rats, see Zhou et al., 2020). Therefore, if mediated by GABA_A_Rs, the longer decay times in KOs would imply abnormal expression of synaptic GABA_A_Rs upon GlyT2 gene deletion. Because of the non-specific action of gabazine at GlyRs (Wang and Slaughter, 2005; Li and Slaughter, 2007; Beato, 2008), the drug cannot unequivocally distinguish between GABA_A_Rs and α_2_GlyRs. We therefore refrained from applying gabazine. Instead, we analyzed sIPSCs from KO principal LSO neurons in the presence of pentobarbital (30 μM Pbt). Pbt slows the decay kinetics at GABA_A_Rs without affecting GlyRs (Apostolides and Trussell, 2013; Moore and Trussell, 2017; Fischer et al., 2019). We found that Pbt did not affect *q* (Figures 3D*a,b*; Table 1). More importantly, Pbt changed neither τ_*w*_, nor τ_1_, nor τ_2_ (Figures 3E,F; Table 1), suggesting that abnormal expression of synaptic GABA_A_Rs does not take place. Rather, our results are suggestive of fetal α_2_GlyRs in KOs, implying disturbed GlyR maturation at LSO neurons and a role of GlyT2 in synapse development (Friauf et al., 1999). The increase in sIPSC τ_*w*_ may also be caused by glycine accumulation in the synaptic cleft, by changes in the nanoarchitecture of pre- and postsynaptic elements, and/or by activation of extrasynaptic GlyRs.

Like in Figure 2C, we again addressed the bias problem associated with the unbalanced approach (KO: 7-100 sIPSCs/neuron, KO + Pbt: 16-145 sIPSCs/neuron) by randomly picking seven sIPSCs for each neuron and subsequently plotting the cumulative distribution (Figure 3E, inset). The results confirmed the above findings that were obtained with unequal numbers of sIPSCs/neuron. Also with this approach Pbt did not change τ_*w*_ in KOs (unpaired 2-tailed *t*-test; *P* = 0.08). Thus, the results confirmed the above findings obtained with different unequal numbers.

### MNTB-LSO Microcircuit and Synaptic Strength Are Severely Impaired in GlyT2 KO Mice

Because of the unchanged quantal size *q* in KOs (Figure 2D*b*; Table 1), the drastically reduced eIPSC amplitudes in KOs cannot be explained by insufficiently filled SVs or fewer GlyR molecules on the subsynaptic side. Rather, the reduced amplitudes may be due to fewer or differently distributed GlyRs on the postsynaptic site or a different morphology of the postsynaptic site in general. They may also be due to fewer axon terminals or release sites per MNTB neuron (lower single fiber strength) and/or fewer neurons converging on a given LSO principal cell (lower input number). We determined both parameters in recordings during which we gradually increased the stimulus amplitude, thereby recruiting individual MNTB fibers step by step. Whereas, eIPSC amplitudes at Ctrl inputs increased in a stepwise manner as a function of stimulus amplitude, KO synapses demonstrated barely any increase. For the representative Ctrl and KO neurons depicted in Figures 3G,H, the input number amounted to 5 and 1, respectively. Mean sample values amounted to 4.5 and 1.3 (Figure 3I*b;* Table 2). The results indicate that a single LSO principal neuron in postnatal day 11 GlyT2 KOs receives input from only ~1 presynaptic MNTB neuron. Compared to the normal situation, where we found a ~4:1 convergence, the input number is reduced by >70%. The maximal stimulation amplitudes were most probably high enough to recruit all inputs. Therefore, it is unlikely that very small inputs with extraordinarily high thresholds were overlooked. The lower input number in KOs was paralleled by a drastically reduced single fiber strength (>5-fold, Figure 3I*a*; Table 2), demonstrating impaired efficacy of the remaining MNTB fiber to inhibit the LSO target neuron. Conjunctively, the reduced input number and the reduced single fiber strength lead to dramatically reduced inhibition in KOs, as evidenced by huge differences in maximal eIPSC amplitudes (~27-fold, Figure 3I*c*; Table 2).

Low spontaneous rates and small sIPSC amplitudes, as we report here (cf. Table 1), can be related to a small readily releasable pool of SVs (*N*_*RRP*_) and/or a low release probability *P*_*v*_ (Kaeser and Regehr, 2014). To address the two possibilities for the MNTB-LSO inputs, we assessed the size of the initial readily releasable pool (*RRP*_1_). For this purpose, we stimulated MNTB axons at 50 Hz in order to exhaust the *RRP* (Figure 3J*a*). Experiments were done at half-maximal stimulus amplitude, thus activating ~2.25 MNTB input neurons per LSO target neuron. Resulting cumulative eIPSC amplitudes, which represent the postsynaptic current evoked by the *RRP* (*I*_*RRP*_), were strikingly smaller in KOs (~5-fold, Figures 3J*b*,**K***a*; Table 3). By implementing *q*, we calculated the number of SVs comprising *RRP*_1_ for each MNTB-LSO connection via $N_{RRP1}=\frac{I_{RRP1}}{q}$. *N*_*RRP*1_in KOs was drastically decreased (>5-fold, Figure 3K*a*; Table 3). Considering that the mean input number was reduced from 4.5 to 1.3 (Table 2), we conclude that the KO MNTB-LSO microcircuit is not only severely impaired by a loss of functional inputs, but also by a smaller *RRP*_1_ in the remaining input of ~1 MNTB neuron. In fact, the mean *N*_*RRP*1_ of a given MNTB input neuron appears to be reduced by ~2/3 (144 SVs/2.25 fibers vs. 27 SVs/1.3 fibers, relating to 64 vs. 21 SVs/fiber).

Having determined *RRP*_1_, we calculated the release probability $P_{v}=\frac{eIPSC_{1}}{I_{RRP_{1}}}$. KO values were almost 2-fold higher than in Ctrls (Figure 3K*b*; Table 3), implying that MNTB-LSO synapses in GlyT2 KOs react to the loss of GlyT2 and ‘try' to compensate the lower input number and reduced *RRP*_1_ by increasing *P*_*v*_. However, the compensation attempts are obviously insufficient (cf. Figures 3A,B*a***,J***a*,*b*).

### During High-Frequency Stimulation, MNTB-LSO Inputs in GlyT2 KOs Depress More Heavily and Display Higher Failure Rates

**Figure 4:**
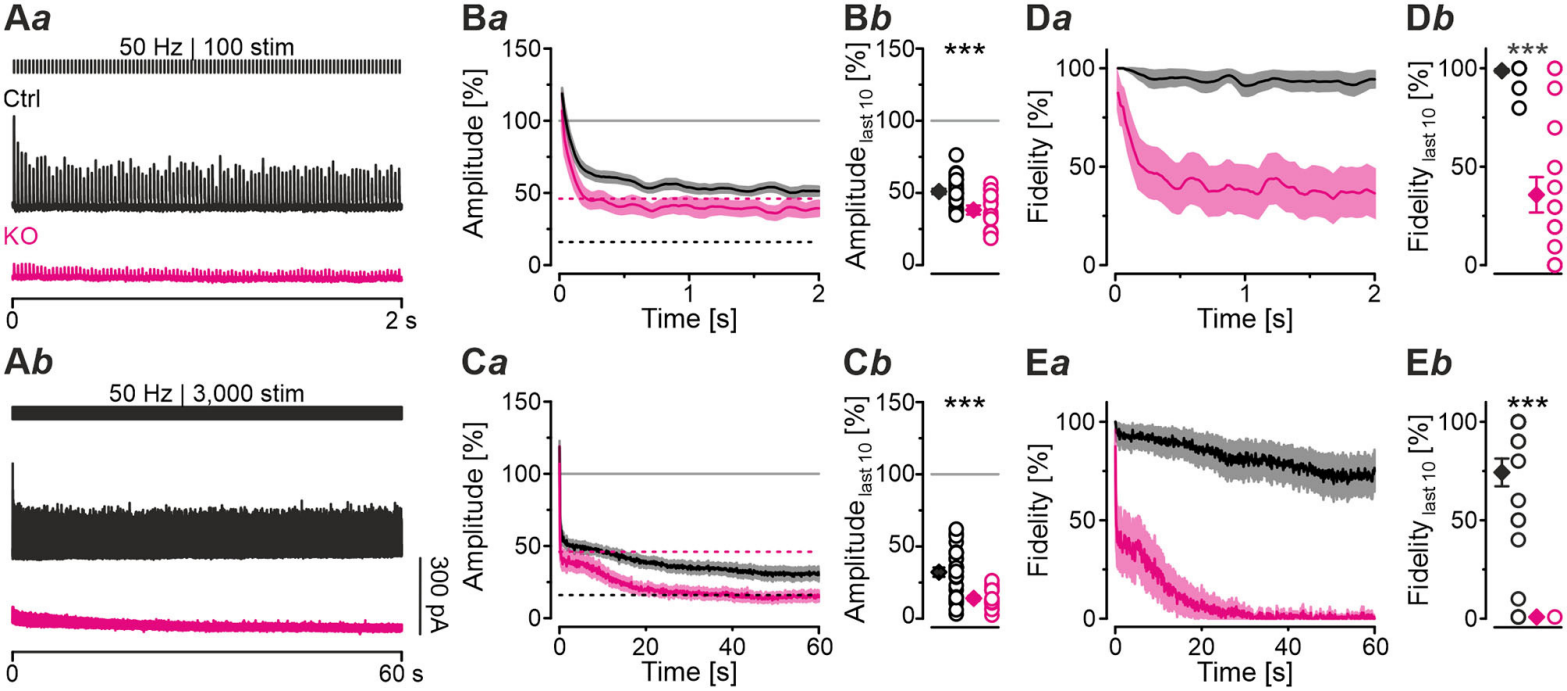
MNTB-LSO synapses in KO mice display stronger synaptic depression and higher failure rate during high-frequency stimulation. **(A)** Current traces from a representative Ctrl and KO neuron, depicting eIPSCs during high-frequency stimulation (= challenge period) lasting 2 s [50 Hz | 100 stim, **(A***a***)**] or 60 s [50 Hz | 3,000 stim, **(A***b***)**]. **(B,C**) Time course of normalized eIPSC sample amplitudes **(B***a*,**C***a***)** during challenge periods lasting 2 s **(B)** or 60 s **(C)**, and statistics at the end of challenge periods **(B***b*,**C***b***)**. Time courses depict weighted moving average (SEM light-shaded). Black and purple dashed lines show mean detection thresholds for Ctrl and KO samples, respectively. Gray horizontal lines show baseline (= 100%). **(D,E)** Same as **(B,C)**, but for fidelity. Details in Table 4. For meaning of ***, see Materials and Methods, Statistics.

Synapses with high *P*_*v*_ values tend to display short-term depression (STD; Xu-Friedman and Regehr, 2004; Regehr, 2012). The higher *P*_*v*_, the more profound is the STD behavior. Because of the higher *P*_*v*_ observed in KOs, we hypothesized that STD in KOs is much more pronounced than in Ctrls. To address the point, we challenged MNTB-LSO inputs with short- or long-lasting high-frequency stimulus trains (50 Hz for 2 s or 60 s). Representative current traces depicting the time course of eIPSC amplitudes are shown in Figures 4A*a,b*. Ctrl sample behavior during 2-s trains was characterized by the typical exponential decline described previously (Figure 4B*a*; cf. Krächan et al., 2017; Brill et al., 2019). At the train's end, eIPSC amplitudes amounted to ~50% of the baseline level (Figures 4B*a,b*; Table 4). KO inputs depressed more heavily. At the train's end, eIPSC amplitudes reached ~40%, significantly lower than the 50% level for Ctrls (Figures 4B*a,b*; Table 4). Nevertheless, the time courses were statistically indistinguishable during the first seven stimuli (equivalent to 120 ms). Thereafter, responses to stimuli 8-20 became significantly lower in KOs in 8 of 13 cases (not shown). Notably, steady-state amplitudes, obtained after <0.5 s, straddled the detection threshold (dashed purple line in Figure 4B*a*) and were thus subthreshold in many cases. When checking fidelity behavior, we found virtually no transmission failures in Ctrls throughout the 2-s train (Figures 4D*a*,*b*; Table 4). In contrast, KO inputs were unable to transmit failure-free, and mean fidelity declined to 36% by the train's end (Figures 4D*a*,*b*; Table 4).

**Table 4:** Synaptic depression and recovery of MNTB-LSO inputs in Ctrl, KO, and ALX during short- and long-lasting challenge | recovery periods.

		**Fidelity [%]**			***P*** **value significance**		
		**Ctrl**	**KO**	**ALX**	**Ctrl vs. KO**	**Ctrl vs. ALX**	**KO vs. ALX**
Challenge	50 Hz | 2 s last 10	99 ± 1 (25)	36 ± 9 (16)	99 ± 1 (14)	1.8 × 10^−7(U)^ ^***^	0.6^(U)^ n.s.	3.0 × 10^−5(U)^ ^***^
	50 Hz | 60 s last 10	74 ± 7 (27)	0 ± 0 (15)	65 ± 11 (15)	5.0 × 10^−7(U)^ ^***^	0.3^(U)^ n.s.	7.2 × 10^−6(U)^ ^***^
		**Amplitudes [%]**			***P*** **value significance**		
Challenge	50 Hz eIPSC_1_	119 ± 4 (27)	107 ± 12 (16)	116 ± 3 (14)	0.4 n.s.	0.6 n.s.	0.5 n.s.
	50 Hz | 2 s last 10	51 ± 2 (27)	39 ± 2 (25)	53 ± 2 (15)	4.8 × 10^−4^ ^***^	0.5 n.s.	4.1 × 10^−4^ ^***^
	50 Hz | 60 s last 10	32 ± 3 (27)	14 ± 2 (15)	28 ± 4 (15)	1.3 × 10^−5^ ^***^	0.4 n.s.	0.007 ^*^
Recovery	1 Hz | 60 s last 10	92 ± 5 (27)	38 ± 4 (16)	100 ± 3 (15)	8.8 × 10^−10^ ^***^	0.2 n.s.	1.6 × 10^−12^ ^***^
*RecovA*	*P* value significance	0.1 n.s.	1.5 × 10^−10^ ^***^	0.9 n.s.	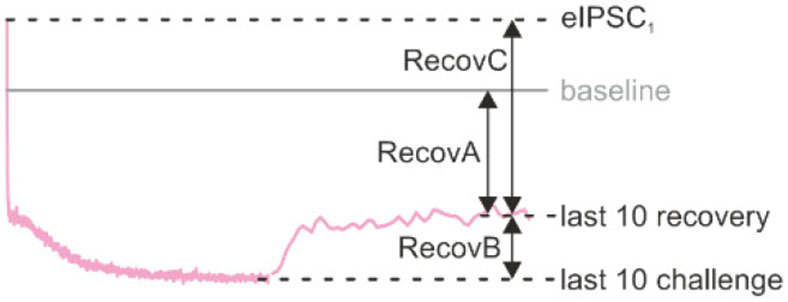		
*RecovB*	*P* value significance	2.4 × 10^−11^ ^***^	1.7 × 10^−4^ ^***^	2.6 × 10^−9^ ^***^			
*RecovC*	*P* value significance	1.3 × 10^−6^ ^***^	4.0 × 10^−5^ ^***^	0.004 ^*^			
*FR* [%]		89 ± 8 (27)	25 ± 5 (16)	105 ± 5 (14)	7.6 × 10^−8^ ^***^	0.1 n.s.	1.3 × 10^−11^ ^***^

Mean values ± SEM of fidelity at end of challenge periods (last 10 eIPSCs of 50 Hz | 2 s or 50 Hz | 60 s), as well as normalized eIPSC amplitudes to 1st stimulus of challenge (eIPSC_1_), at end of challenge (50 Hz | 2 s or 50 Hz | 60 s), and at end of recovery periods (1 Hz | 60 s) for Ctrl, KO, and ALX samples (cf. Figures 4, 6, 8). Also depicted are P values, significance levels, and n. Statistics: Ctrl vs KO, Ctrl vs. ALX, and KO vs. ALX: unpaired 2-tailed t-test or Mann Whitney U-test ^(U)^; Šidák correction (k = 2 or 5). Recovery behavior was assessed by four means (cf. inset; see also Brill et al., 2019): RecovA: last 10 eIPSCs of recovery vs. baseline (= 100%); RecovB: last 10 eIPSCs of recovery vs. last 10 eIPSCs of preceding challenge; RecovC: last 10 eIPSCs of recovery vs. eIPSC_1_ of preceding challenge; Fractional recovery FR = $\frac{last\;10\;eIPSCs\;recovery-last\;10\;eIPSCs\;challenge}{baseline-last\;10\;eIPSCs\;challenge}$. Statistics for RecovA-C: paired two-tailed t-test. Šidák correction (k = 5). Statistics for FR: unpaired two-tailed t-test. Šidák correction (k = 2). Recordings were done with 2 mM [Cl^−^]_i_. For meaning of

*, **, ***, *see Materials and Methods, Statistics*.

To further analyze the synaptic performance in the absence of GlyT2, we challenged MNTB-LSO inputs more heavily in long-lasting trains (60 s), during which SV recycling via endocytosis and neurotransmitter re-uptake, in case of the glycinergic MNTB-LSO synapses by GlyT2, is very likely (Ryan et al., 1993; de Lange et al., 2003; Rizzoli, 2014; Watanabe et al., 2014). By the end of such 60-s trains, Ctrls had depressed to ~30% and KOs >2-fold more (to 14%, Figures 4C*a*,*b*; Table 4). In terms of absolute amplitudes, 32% and 14% corresponded to 110 pA and 8 pA, respectively. Note that in KOs, eIPSC peak amplitudes of ≤ 24 pA were below the detection threshold. Consequently, eIPSC amplitudes of 8 pA were associated with a fidelity behavior of 0%, i.e., in KOs with a virtually complete collapse of transmission during the 2nd half of the train (Figures 4E*a*,*b*). By contrast, the mean value of 110 pA in Ctrls was weigh above the detection threshold, resulting in 74% fidelity (Figures 4C*a***,E***a*,*b;* Table 4).

The curves depicted in Figure 4C*a* revealed another interesting aspect, namely a profound decrease in eIPSC amplitudes in KOs after ~8 s, thereby ending a relatively long period (~4 s) of steady-state amplitudes that was much less pronounced in Ctrls. This time course was also prominent in the fidelity curve (Figure 4E*a*). Taken together, STD during 2-s trains is stronger in KOs; the ratio 51/39% corresponds to a 1.3-fold higher effect. STD is even more profound in 60-s trains, with the ratio 32/14% corresponding to a 2.3-fold higher effect (Figure 4C*b;* Table 4). Concerning fidelity, the differences are 2.75-fold and ∞-fold, respectively (Figures 4D*b*,**E***b;* Table 4).

### Computational Modeling Captures Experimental Data Very Well and Reveals Starkly Impaired Replenishment Capability in GlyT2 KOs

**Figure 5:**
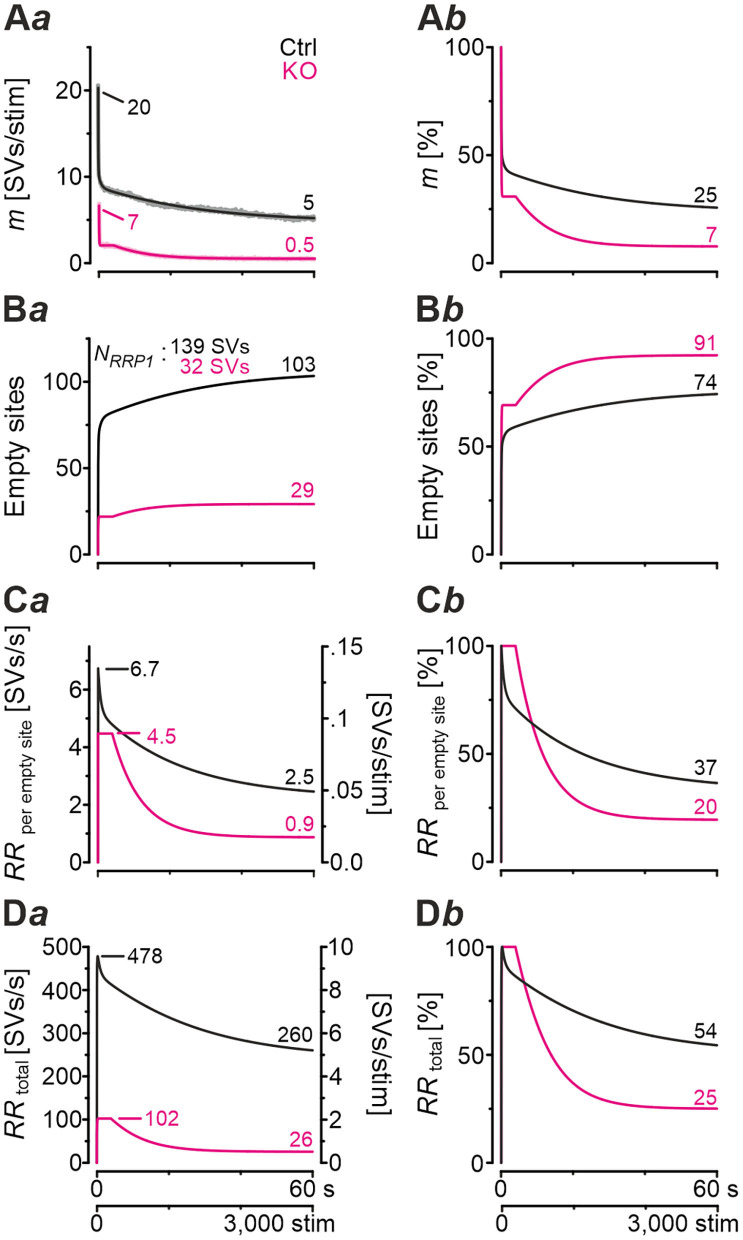
Docking site model captures experimental data very well and points to strikingly impaired replenishment in KO mice. **(A–D)** Time courses of quantal content *m* **(A)**, number of empty release sites **(B)**, replenishment rate per empty site [*RR*_peremptysite_, **(C)**], and total *RR* [*RR*_total_, **(D)**] during sustained stimulation (50 Hz | 60 s) of Ctrl and KO inputs **(A***a*–**D***a***)**. For better comparison across genotypes, normalized data are also presented **(A***b***–D***b***)**. Dark-shaded curves **(A***a***)** represent modeled results and light-shaded curves empirical results (cf. Figure 4C*a*). Note almost perfect congruency of modeled and empirical curves. Experimental data for m values were 17 and 2 SVs/stim for Ctrls and KOs, respectively, and modeling revealed 20 and 7 SVs/stim, respectively.

In a next step, we applied a double-exponential analytical model to the depression curves obtained during 50 Hz | 60 s challenge (Equation 3; 18 Ctrl, 9 KO inputs). We did the computational modeling in an SV-based manner, which enables a direct comparison of the synaptic performance across synapses. We converted eIPSC amplitudes into quantal content *m*. As obvious from Figure 5A*a*, the model captured the experimental data very well, including the 4-s steady state period for the KOs. In Ctrls, *m* declined from an initial value of 20 SVs/stim to 5 SVs/stim at the train's end, i.e., to 25% (compare with 32% value in Figure 4C*b*). In KOs, *m* declined from 7 to 0.5 SVs/stim, i.e., to 7% (compare with 14% in Figure 4C*b*). Thus, the decline at GlyT2-lacking inputs is 3.6-fold more profound than at inputs with functional GlyT2 (Figure 5A*b*). Modeling also revealed a very rapid decline of *m* in KOs, reaching ~2 SVs/stim at stimulus #22 and remaining at this level until stimulus #204, i.e., for ~3.6 s. In Ctrls, modeling demonstrated an *m* value of ~10 SVs/stim at stimulus #22, declining further to 8.3 SVs/stim until stimulus #204. In other words, it highlights the transient plateau in KOs from ~0.4 s until ~4.0 s that is absent in Ctrls (cf. Figure 4C*a* for different cohorts).

Computational modeling also enabled us to calculate the replenishment rate (*RR)*, i.e., the frequency at which empty release sites get reoccupied. We assumed that all release sites are occupied at the beginning of the challenge period, i.e., the number of empty sites is 0 (*M* = *N*_*RRP*1_; Figure 5B*a*). Moreover, the number of occupied sites is equivalent to the *N*_*RRP*1_ which comprised 139 SVs in Ctrls and 32 SVs in KOs according to the model (Table 3). The model determined a *P*_*v*_ of 14% in Ctrls and 22% in KOs and assumes that these values remain constant throughout the experiment. In response to stimuli #1-10, and in parallel with the profound STD, the number of occupied sites decreased rapidly from 139 to 71 in Ctrls and from 32 to 11 in KOs (not shown). The decrease was associated with an increase of empty sites (Ctrl: 68; KO: 21; Figure 5B*a*). After 50 stimuli, there are 77 and 22 empty sites in Ctrls and KOs, respectively, and at the end of the trains, numbers are 103 and 29, corresponding to 74 and 91% of *N*_*RRP*1_ (Figures 5B*a*,*b*). Thus, KO inputs display an almost totally depleted *RRP*, whereas the number of occupied sites is kept at 26% in the presence of functional GlyT2s.

Next, we determined the replenishment rate *RR*_peremptysite_ through modeling (Figures 5C*a*,*b*). Whereas, the maximum value was 6.7 SVs/s per empty site in Ctrls, it was 4.5 in KOs (at stimulus #4 and stimuli #4-200, respectively). Values at the train's end were 2.5 and 0.9, relating to 37% and 20% of the maximal value, respectively. Finally, we determined the total *RR*, which calculates to

$$\begin{array}{l} RR_{total}=\#empty\;sites*RR_{per\;empty\;site} \end{array}$$

KO MNTB-LSO inputs achieve a maximal *RR*_*total*_ of 102 SVs/s during the transient plateau phase, whereas Ctrls reoccupy empty sites at a maximal rate of 478 SVs/s, i.e., at an almost 5-fold higher rate (Figures 5D*a*,*b*). By the end of the train, the difference has amounted to 10-fold (26 vs. 260 SVs/s), which relates to 25% and 54% of the maximal capacity. A steep decline of *RR*_*total*_, particularly during seconds 4–20, highlights the SV replenishment defects during sustained high-frequency stimulation that occur after GlyT2 loss (Figure 5D*b*).

### Severely Impaired Recovery From Synaptic Depression in GlyT2 KOs

**Figure 6:**
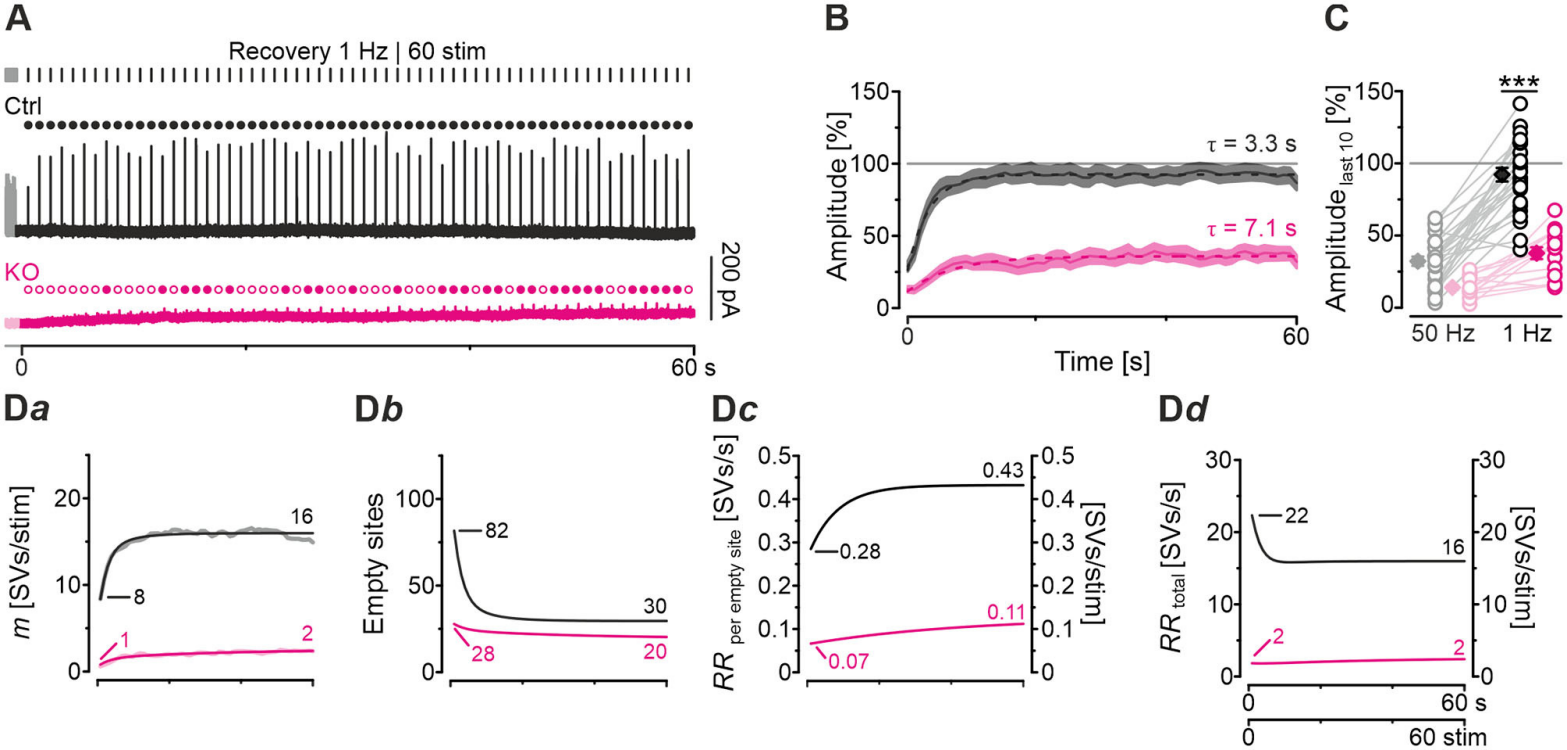
Recovery from synaptic depression is severely impaired at KO synapses. **(A)** Current traces from a representative Ctrl and KO neuron, depicting eIPSCs during a 60-s recovery period (1 Hz | 60 stim) following challenge (50 Hz | 3,000 stim | 60 s). For clarity, the last second of each preceding challenge period is also illustrated (light-shaded). Open and filled circles mark eIPSC amplitudes below and above detection threshold, respectively. **(B)** Time course of normalized sample amplitudes during recovery (SEM light-shaded). Time courses depict weighted moving average. Monoexponential fits for Ctrl and KO are illustrated by black and purple dashed lines, respectively. Gray horizontal line indicates baseline. **(C)** Statistics at the end of challenge and recovery periods (50 and 1 Hz; mean values of last 10 eIPSCs; paired data). Details and further statistics in Table 4. **(D)** Docking site model. Time course of *m* **(D***a***)**, number of empty sites **(D***b***)**, *RR*_peremptysite_ **(D***c***)**, and *RR*_total_ **(D***d***)** during recovery period. Dark and light-shaded curves **(D***a***)** represent modeled data and empirical data, respectively [cf. **(B)**]. For meaning of ***, see Materials and Methods, Statistics.

So far, our results have demonstrated drastically impaired replenishment capabilities at KO synapses, particularly during high-frequency challenge. Whereas, Ctrl MNTB-LSO inputs replenish 260 SVs/s at the end of the challenge period, this capacity is reduced to a 10th at KO inputs (Figure 5D*a*). We wondered whether replenishment during periods with no stimulation, or only little, would enable the inputs to recover completely from synaptic depression. To tackle the question, we applied a 60-s recovery period after the 60 s | 50 Hz challenge period during which we activated the inputs with 1-Hz test stimuli to assess recovery kinetics. Representative current traces from a Ctrl and a KO input are illustrated in Figure 6A. Ctrl eIPSCs recovered quickly during the 1st 4 s, after which steady-state amplitudes were obtained. In contrast, the KO remained depressed, and 32 of 60 responses remained below the detection level and were thus failures. The first suprathreshold response occurred after 8 s. In striking contrast, no failure was present in the Ctrls.

The recovery behavior of the samples could be described by a monoexponential function for both genotypes with a mean time constant τ of 3.3 s and 7.1 s for Ctrls and KOs, respectively (Figure 6B). These values compare to τ_fast_ and τ_*slow*_ values of ~6.7 s and ~1 min reported for hippocampal Schaffer collateral synapses (Gabriel et al., 2011). Like in a previous paper (Brill et al., 2019), we quantified recovery in several ways (see Methods and inset in Table 4). We normalized the eIPSC amplitudes to the baseline level (=100%), which was obtained as the arithmetic mean of 40–60 eIPSC peak amplitudes obtained at 1 Hz. Most often, the 1st eIPSC in the challenge trains exceeded this 100% value, which we previously alluded to as ‘manic behavior' (cf. Friauf et al., 2015). Regarding *RecovA*, Ctrl inputs recovered to ~90%, thus becoming indistinguishable from the baseline level (Figures 6B,C; Table 4). In contrast, KO inputs recovered incompletely to only ~40%. *RecovB* analysis showed significant amplitude increases for Ctrls (92 vs. 32% = ~3-fold) as well as KOs (38 vs. 14% = ~3-fold). For *RecovC*, recovery was incomplete in both genotypes, i.e., overshooting ‘manic' eIPSC_1_ amplitudes of >100% were not regained (Ctrl: 92 vs. 119%; KO: 38 vs. 107%). In a fourth and final step, we assessed the fractional recovery (*FR*; Table 4). In Ctrls, *FR* calculated to ~90%, whereas it was significantly smaller in KOs (25%). Once again, the data imply severely impaired SV replenishment upon GlyT2 gene deletion.

### Computational Modeling of Recovery Behavior

Modeling captured the empirically obtained recovery curves very well and demonstrated that KOs release only 2 SVs/stim at the end of the recovery period, whereas Ctrls release 16 SVs/stim (Figure 6D*a*). Moreover, modeling revealed 82 empty sites after 1 s into recovery in Ctrls (Figure 6D*b)*. As 103 empty sites were present at the end of 50-Hz challenge (Figure 5B*a*), Ctrls reoccupy 21 sites within the 1st s of recovery. In contrast, only 1 empty site becomes reoccupied in KOs (28 empty sites after 1 s into recovery vs. 29 at end of preceding challenge period). During the remainder of the 60-s recovery period, Ctrls manage to reoccupy 61% of the remaining empty sites (50 of 82), whereas KOs replenish only 29% (8 of 28). The initial *RR*_peremptysite_ is 0.07 SVs/s in KOs, thus reaching only 25% of the 0.28 value in Ctrls (Figure 6D*c*). Throughout the recovery period, *RR*_peremptysite_ increases for both genotypes (KO: from 0.07 to 0.11 SVs/s, ~1.6-fold; Ctrl: from 0.28 to 0.43 SVs/s, ~1.5-fold; Figure 6D*c*). As Ctrls display a ~4-fold higher *RR* than KOs, their empty sites become reoccupied more efficiently and, consequently, *RR*_*total*_ declines from 22 to 16 SVs/s. In contrast, it stays at 2 SVs/s throughout the recovery period in KOs (Figure 6D*d*). Collectively, our results show drastically impaired replenishment of glycinergic SVs upon GlyT2 gene deletion. Nevertheless, and unexpectedly, a complete depletion of synaptic transmission does not occur at KO MNTB-LSO inputs, even under sustained challenging.

### Acute Pharmacological Blockade of GlyT2 Has Only Minor Effects on Short-Lasting Synaptic Transmission

**Figure 7:**
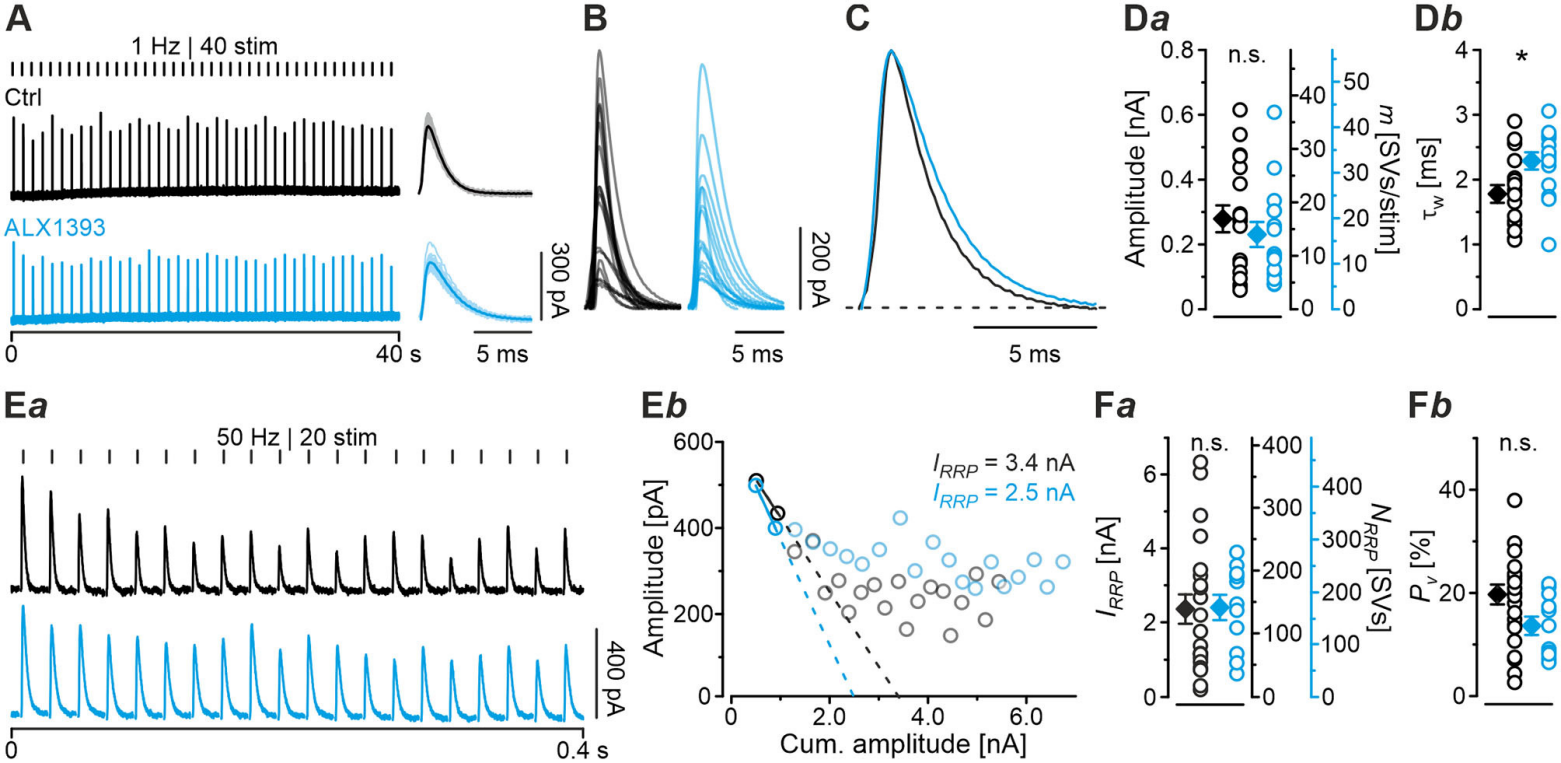
Acute pharmacological blockade of GlyT2 leads to prolonged eIPSC decay time. **(A)** Current traces depicting eIPSCs from a representative Ctrl neuron (black) and a neuron in the presence of a specific GlyT2 antagonist (2 μM ALX1393, cyan). Left part shows raw traces (1 Hz | 40 stim), right part shows individual eIPSCs and graphical mean (light- and dark-shaded). **(B)** Overlay of mean eIPSCs for Ctrl and ALX samples. **(C)** Peak-scaled eIPSCs (sample means), depicting kinetics for Ctrl and ALX. Dashed line indicates resting level. **(D)** Statistics for amplitude and *m* **(D***a***)** as well as τ_w_ **(D***b***)**. **(E)** Estimation of *I*_*RRP*_. Representative current traces **(E***a***)**, depicting 20 eIPSCs during 50 Hz stimulation for a Ctrl and an ALX neuron. Corresponding Elmqvist-Quastel plots **(E***b***)**. **(F)** Statistics for *I*_*RRP*_ and *N*_*RRP*_**(F***a***)** and *P*_*v*_ **(F***b***)**. Y-axes for *N*_*RRP*_ vary slightly due to small differences in *q*. Details in Table 3. For meaning of *, see Materials and Methods, Statistics.

Having demonstrated several severe deficits in glycinergic neurotransmission at KO MNTB-LSO inputs, we wondered about effects of acute GlyT2 blockade. For this purpose, we turned to pharmacological experiments in which we applied the selective GlyT2 antagonist ALX1393 into the ACSF (Gether et al., 2006; Dohi et al., 2009; Zeilhofer et al., 2018). Of six basic synaptic properties analyzed, five remained unchanged, namely eIPSC amplitude and quantal content *m* (Figure 7D*a*), *I*_*RRP*_ and *N*_*RRP*_(Figures 7E*a,b*,**F***a*), and *P*_*v*_ (Figure 7F*b*; see also Table 3). A statistically significant difference occurred only for τ_*w*_, which was 1.3-fold prolonged (Figure 7D*b;* Table 3). Together, the results show that acute GlyT2 blockade does not result in major defects in basic glycinergic neurotransmission at MNTB-LSO inputs. Rather, the results imply that the inputs can utilize glycine sources that are independent of GlyT2 re-uptake activity. One possibility are large reserve pools, other glycine transporters, or powerful biosynthesis pathways (see below and Discussion).

### Acute Pharmacological Blockade of GlyT2 Does Not Affect Synaptic Depression During High-Frequency Stimulation but Slows Down Recovery From Depression

**Figure 8:**
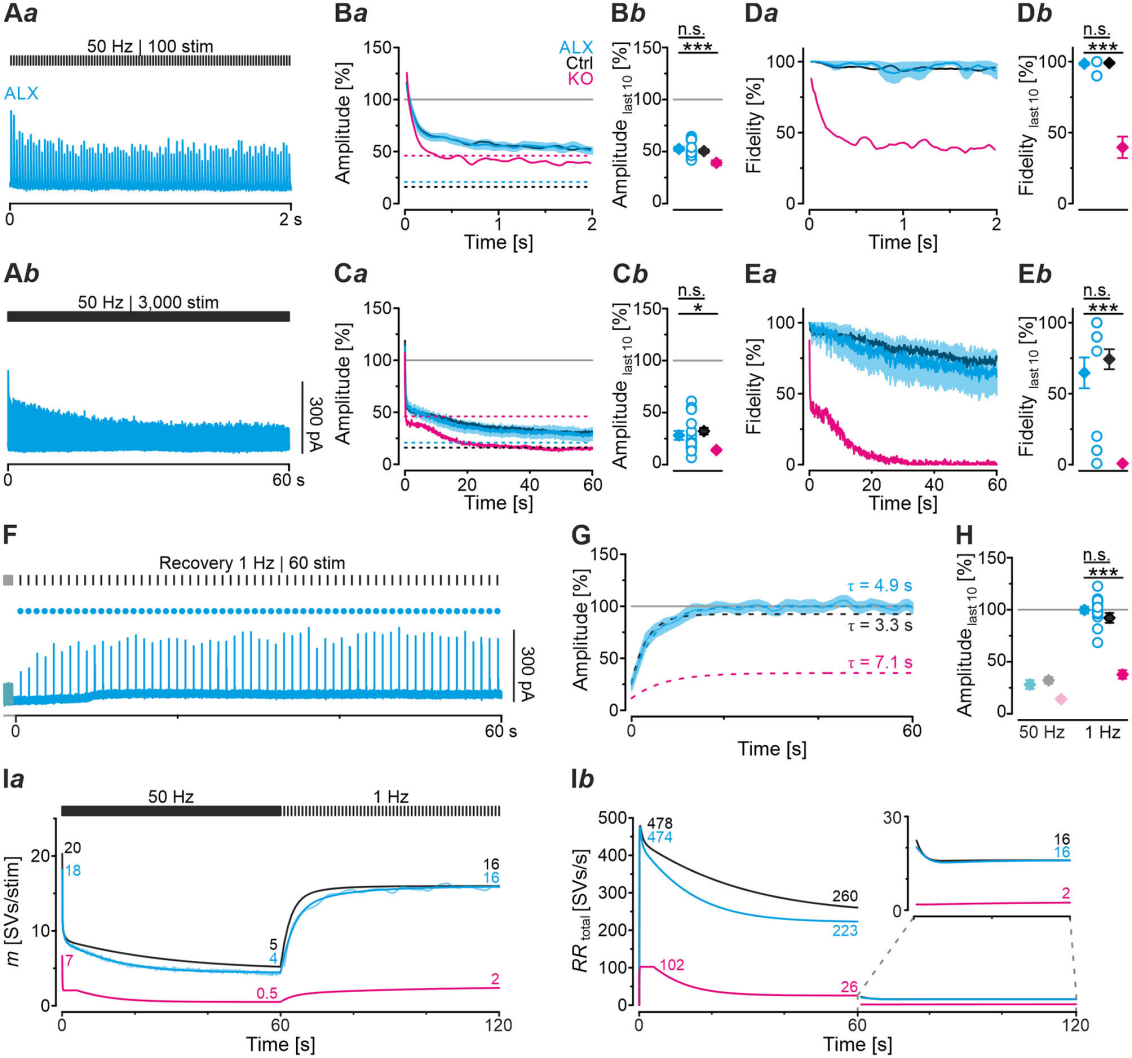
Acute pharmacological blockade of GlyT2 does not affect synaptic depression during high-frequency stimulation, but slows recovery from depression. **(A)** Current traces from a representative ALX neuron, depicting eIPSCs during high-frequency challenge lasting 2 s [50 Hz | 100 stim, **(A***a***)**] or 60 s [50 Hz | 3,000 stim, **(A***b***)**]. **(B,C)** Time course of normalized amplitudes **(B***a*,**C***a***)** during challenge periods lasting 2 s **(B)** and 60 s **(C)** for ALX, Ctrl, and KO samples, and statistics at the end of challenge periods **(B***b*,**C***b***)**. Time courses depict weighted moving average (SEM light-shaded). Black, purple, and cyan dashed lines indicate mean detection thresholds for Ctrl, KO and ALX samples, respectively. Gray horizontal line indicates baseline. **(D,E)** Same as **(B,C)**, but for fidelity. See Figure 4 for further results from Ctrl and KO samples. **(F)** Current trace for a representative ALX neuron, depicting recovery (1 Hz | 60 stim |60 s) following challenge (50 Hz | 3,000 stim | 60 s). The last second of the preceding challenge period is also illustrated (light-shaded). Filled circles indicate amplitudes above detection threshold. **(G)** Time course of normalized amplitudes for ALX sample during recovery (weighted moving average, SEM light-shaded). Cyan, black, and purple dashed lines indicate monoexponential fits for ALX, Ctrl, and KO, respectively. Gray horizontal line indicates baseline. **(H)** Statistics for the end of recovery periods [see **(C***b***)** and Figure 6 for further results]. Details and further statistics in Table 4. **(I)** Docking site model for challenge and recovery (50 and 1 Hz). Time course of *m* **(I***a***)** and *RR*_total_ **(I***b***)** for ALX, Ctrl, and KO. Dark and light-shaded curves in **(I***a***)** represent modeled data and empirical data, respectively [cf. **(C***a*,**G)**]. For meaning of *, ***, see Materials and Methods, Statistics.

The result of virtually unchanged basic neurotransmission upon acute GlyT2 blockade suggested to us that the various SV pools might be sufficiently large to enable synaptic inhibition at low-frequency stimulation, at least for several seconds (1 Hz | 40 s). We therefore employed high-frequency stimulation as above (cf. Figure 4) to challenge the inputs more severely. A representative current trace depicting the time course of eIPSC amplitudes in the presence of ALX during 50 Hz | 2 s challenge is depicted in Figure 8A*a*. Sample data showed that eIPSC amplitudes and fidelity behavior were statistically indistinguishable from Ctrls (Figures 8B*a,b*,**D***a,b;* Table 4). In contrast, comparison between ALX and KO samples showed more pronounced STD strength and low fidelity levels in KOs (~1.4- vs. ~2.8-fold; Figures 8B*b***,D***b*; Table 4).

Because robust and virtually undisturbed transmission occurred at ALX inputs during 50 Hz | 2 s-trains, we wondered whether sustained stimulation in longer trains would fatigue the synapses. We therefore applied 50 Hz | 60 s-trains and, unexpectedly, found again no significant difference to their Ctrl counterparts (Figures 8A*b*,**C***a,b*,**E***a,b*; Table 4, 28 vs. 32%). Compared to KOs, however, ALX inputs did display 2-fold higher eIPSC amplitudes at the end of challenge and a tremendously higher fidelity. Thus, the effects of acute GlyT2 blockade (ALX) appear to differ considerably from chronic effects (KOs) at MNTB-LSO inputs.

As ALX and Ctrl inputs behaved similarly during high-frequency challenge, we reasoned that subsequent recovery behavior might also be similar. Indeed, recovery levels were statistically indistinguishable, amounting to 100 and 92%, respectively (Figures 8G,H; Table 4). *FR* values for ALX inputs were 105%, implying virtually complete recovery (*FR* for Ctrls: 89%; no significant difference). Recovery was ~1.5-fold slower in ALX than in Ctrls (τ: 4.9 s vs. 3.3 s; Figure 8G). Compared to KOs, ALX inputs recovered 2.6-fold more and *FR* was >4-fold higher (Figures 8G,H; Table 4).

Computational modeling confirmed the striking similarities in depression behavior between ALX and Ctrls and also the slower recovery in ALX (Figure 8I*a*). Moreover, modeling total replenishment activity (*RR*_*total*_) during the recovery period revelead virtually no difference between ALX and Ctrls (Figure 8I*b, inset*). The latter finding was in stark contrast to the KO scenario, where *RR*_*total*_ during the recovery period was 8-fold lower (2 vs. 16 SVs/s). In summary, acute GlyT2 inactivation hardly affects glycinergic transmission at MNTB-LSO inputs. Again, the results corroborate the conclusion that these inputs manage to maintain proper function for tens of seconds despite the lack of GlyT2, and they point to glycine sources other than those supplied by GlyT2 re-uptake.

### Even During Sustained Stimulation With 30,600 Stimuli, Pharmacological GlyT2 Blockade Impairs Resilience of MNTB-LSO Inputs Only Slightly

**Figure 9:**
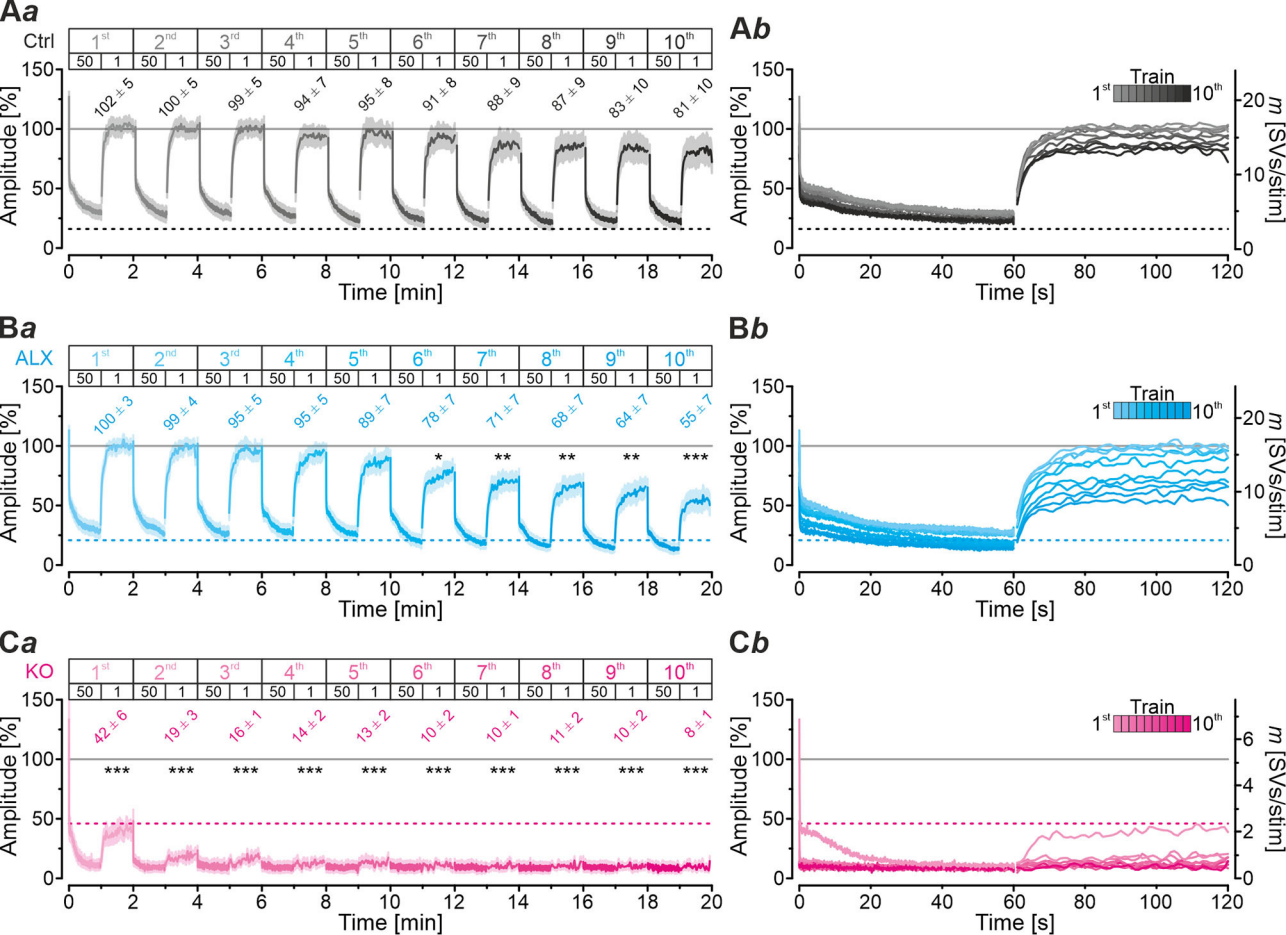
Acute blockade of GlyT2 impairs resilience of MNTB-LSO inputs only slightly during marathon experiments. **(A–C)** Time course of normalized amplitudes during 10 challenge | recovery trains for Ctrl **(A***a***)**, ALX **(B***a***)**, and KO samples **(C***a***)** as well as time-expanded close-ups of the 10 trains **(A***b*–**C***b***)**. Additional Y-axes for *m* in **(A***b*–**C***b***)**. Trains 1–10 are color-coded from light to dark. Time courses depict weighted moving average (SEM light-shaded). Asterisks indicate significance levels for *RecovA* (see methods and Table 4 for details). Numbers refer to amplitudes ± SEM at end of recovery periods. Details in Table 5. For meaning of *, **, ***, see Materials and Methods, Statistics.

Robustness and resilience of MNTB-LSO inputs during sustained high-frequency stimulation are hallmarks of this synapse type (Krächan et al., 2017; Brill et al., 2019). To our surprise. ALX-treated synapses were almost as resilient as untreated synapses during a 50-Hz | 60-s train (cf. Figure 8I), questioning that GlyT2 activity is *the* major player for glycine replenishment at MNTB-LSO synapses. In a final experimental approach, we performed ultralong experiments and offered 50 Hz | 60 s challenge and 1 Hz | 60 s recovery trains 10 times in a row. Such experiments lasted 20 min and comprised 30,600 stimuli. We therefore refer to them as 'marathon-experiments' (Kramer et al., 2014). The results emphasize the resilience and robustness of MNTB-LSO inputs (Figures 9A*a*,*b*). Although depression levels were pronounced in Ctrls at the end of the 10 individual challenge trains, becoming stronger over time and ranging from 30% in the 1st train to 21% in the 10th train, eIPSC amplitudes increased robustly during each subsequent recovery period, namely from a maximal value of 102% in the 1st train to a minimal value of 81% in the 10th train (Figure 9A*a*). In none of the 10 trains was the final amplitude statistically distinguishable from the baseline, i.e., *RecovA* was always complete (cf. Figure 9A*a*; Table 5). Moreover, *N*_*RRP*_ declined only moderately across challenge trains as evidenced by the fact that the lowest value (97 SVs at beginning of 9th train) was still 70% of *N*_*RRP*1_ (Table 5).

**Table 5:** Synaptic depression and recovery of MNTB-LSO inputs in Ctrl, KO, and ALX during ten challenge | recovery trains.

									***RecovA***	
**Train**		**N**_****RRP****_ **[SVs]**			**Amplitudes**_****last10****_ **[%]**			***P*** **value significance**		
		**Ctrl**	**KO**	**ALX**	**Ctrl**	**KO**	**ALX**	**Ctrl**	**KO**	**ALX**
1	Challenge	139	32	155	30 ± 4	11 ± 2	28 ± 4			
	Recovery				102 ± 5	42 ± 6	100 ± 3	0.7 n.s.	7.1 × 10^−6^ ^***^	0.9 n.s.
2	Challenge	110	11	136	30 ± 4	10 ± 2	27 ± 4			
	Recovery				100 ± 5	19 ± 3	99 ± 4	0.9 n.s.	9.5 × 10^−9^ ^***^	0.8 n.s.
3	Challenge	108	5	124	29 ± 4	11 ± 1	23 ± 3			
	Recovery				99 ± 5	16 ± 1	95 ± 5	0.9 n.s.	1.8 × 10^−8^ ^***^	0.4 n.s.
4	Challenge	113	n.d.	121	27 ± 4	8 ± 1	26 ± 4			
	Recovery				94 ± 7	14 ± 2	95 ± 5	0.4 n.s.	7.9 × 10^−9^ ^***^	0.3 n.s.
5	Challenge	109	n.d.	126	23 ± 4	9 ± 2	26 ± 4			
	Recovery				95 ± 8	13 ± 2	89 ± 7	0.6 n.s.	2.3 × 10^−8^ ^***^	0.1 n.s.
6	Challenge	110	n.d.	129	24 ± 4	10 ± 2	16 ± 3			
	Recovery				91 ± 8	10 ± 2	78 ± 7	0.3 n.s.	2.2 × 10^−9^ ^***^	4.8 × 10^−3^ ^*^
7	Challenge	108	n.d.	73	24 ± 5	7 ± 2	18 ± 3			
	Recovery				88 ± 9	10 ± 1	71 ± 7	0.2 n.s.	1.4 × 10^−10^ ^***^	4.8 × 10^−4^ ^**^
8	Challenge	113	n.d.	66	21 ± 4	8 ± 1	16 ± 3			
	Recovery				87 ± 9	11 ± 2	68 ± 7	0.1 n.s.	2.4 × 10^−9^ ^***^	3.4 × 10^−4^ ^**^
9	Challenge	97	n.d.	73	24 ± 5	9 ± 2	14 ± 3			
	Recovery				83 ± 10	10 ± 2	64 ± 7	0.1 n.s.	5.6 × 10^−9^ ^***^	2.2 × 10^−4^ ^**^
10	Challenge	106	n.d.	71	21 ± 5	7 ± 2	14 ± 3			
	Recovery				81 ± 10	8 ± 1	55 ± 7	0.1 n.s.	1.9 × 10^−10^ ^***^	2.8 × 10^−5^ ^***^

Mean values ± SEM of normalized eIPSC amplitudes at end of ten 60-s challenge | 60-s recovery trains (last 10 eIPSCs) for Ctrl, KO, and ALX samples (n = 18, 15, 9; cf. Figure 9). Also depicted are P values and significance levels. Statistics: RecovA: last 10 eIPSCs recovery vs. baseline (= 100%; cf. Table 3 inset), paired two-tailed t-test. Šidák correction (k = 10). Recordings were done with 2 mM [Cl^−^]_i_. n.d. not determined. For meaning of

*, **, ***, *see Materials and Methods, Statistics*.

In the presence of ALX, synaptic depression amounted to 28% at the end of the 1st train and to 14% at the end of the 10th train (Figures 9B*a,b*). The performance was thus similar to that of Ctrls. However, eIPSCs became subthreshold during the 6th train and in subsequent challenge periods, i.e., depression became more pronounced in the 2nd half of the marathon-experiment. Recovery at ALX inputs was initially characterized by prominent amplitude increases to 100% (1st train), yet only 55% were achieved at the end of the 10th train (Figure 9B*a*). Recovery levels remained significantly lower than the baseline in five cases (trains 6–10), implying less effective replenishment than in Ctrls, and demonstrating the requirement of GlyT2 for efficient refilling of empty SVs in the long run. Finally, *N*_*RRP*_ declined substantially after the 6th train, such that the lowest value (66 SVs at beginning of 8th train) was 46% of *N*_*RRP*1_ (Table 5).

In stark contrast to ALX inputs, KO inputs were totally unable to perform reliably during marathon-experiments. They depressed very heavily, with STD levels ranging from 11% (1st train) to 7% (10th train; Figures 9C*a*,*b*; Table 5). This led to almost 100% failures, equivalent to a virtually complete collapse of synaptic transmission. Considerable amplitude regrowth occurred only during the 1st recovery period. However, in none of the 10 trains was the recovery strong enough to become statistically indistinguishable from the baseline (range: 42% in 1st; 8% in 10th train; Figure 9C*a*; Table 5). In addition, *N*_*RRP*_ declined drastically during early trains. At the beginning of the 3rd train, it was only 16% of *N*_*RRP*1_ (5 vs. 32 SVs; Table 5). Collectively, our results demonstrate a crucial functional dependence of MNTB-LSO inputs on ongoing GlyT2 activity if they are activated with a multitude of stimuli at high frequency in ultralong epochs. Upon pharmacological GlyT2 blockade, impaired recovery from synaptic depression becomes statistically significant not before >18,000 stimuli have been applied over a period of 11 min. In contrast, GlyT2 gene deletion results in much more severe defects, implying chronic changes that are absent in acute pharmacological experiments.

### Quantal Size Appears to Be Unchanged During Marathon-Experiments, Even in KOs

**Figure 10:**
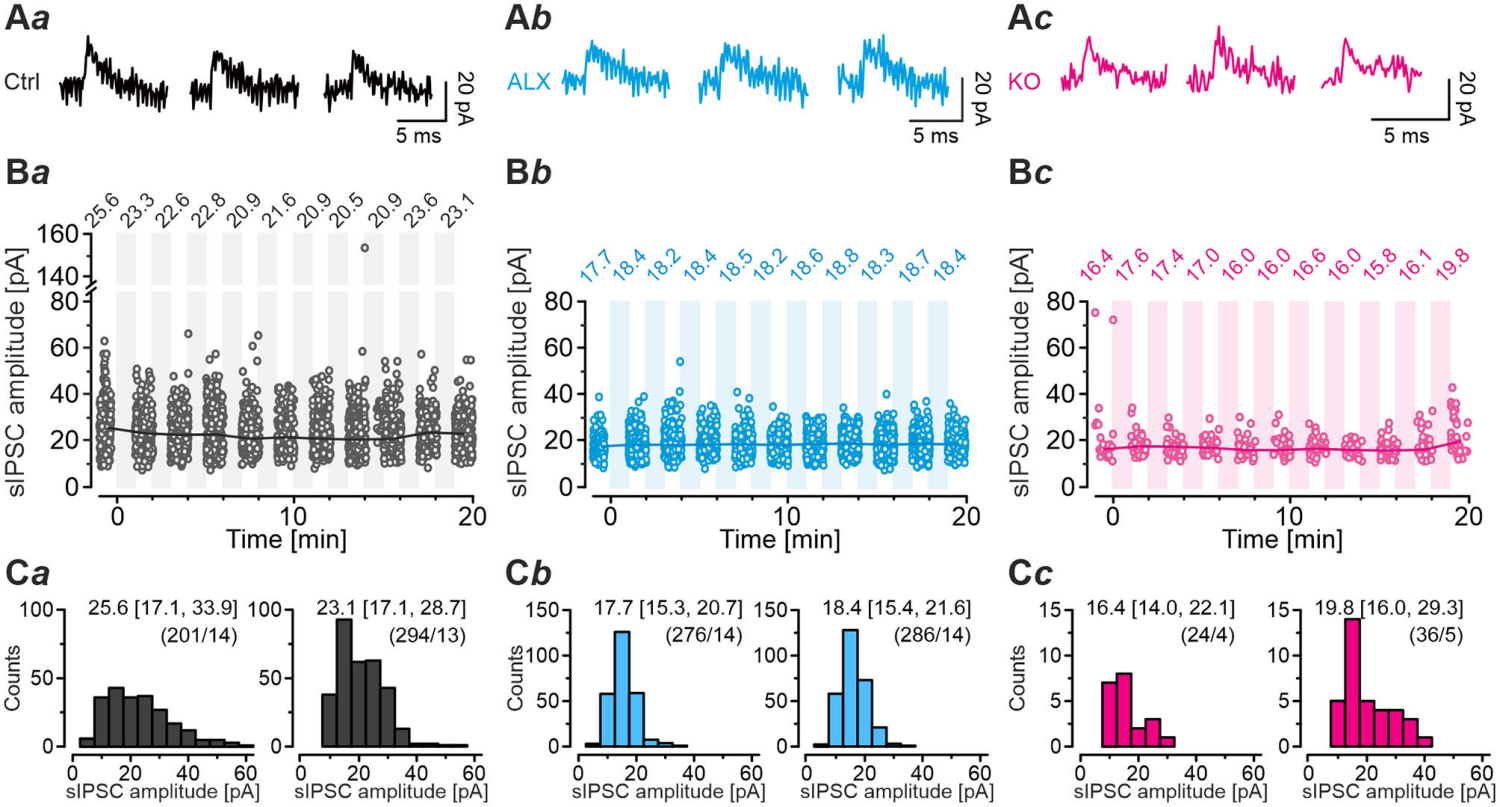
Quantal size remains unchanged during marathon experiments. **(A)** Individual sIPSCs from Ctrl **(A***a***)**, ALX **(A***b***)**, and KO neurons **(A***c***)** during normalization period (left), 5th recovery period (middle), and 10th recovery period (right). **(B)** sIPSC amplitudes (dots) of the Ctrl **(B***a***)**, ALX **(B***b***)**, and KO samples **(B***c***)** during normalization and each recovery period. Circles indicate individual sIPSC amplitudes, lines and numerical values above circles indicate median values. Shaded areas mark challenging periods during which sIPSCs were not evaluated. The analysis captured mIPSCs and multivesicular release, including action potential-triggered release. Number of sIPSCs and neurons: Ctrl, 3030/15; ALX, 3114/17; KO: 351/8. **(C)** Distribution histograms of sIPSC peak amplitude of the Ctrl **(C***a***)**, ALX **(C***b***)**, and KO samples **(C***c***)** for normalization period (left) and 10th recovery period (right). Numbers depict median and the interquartile range in square brackets. Values in round brackets depict number of sIPSCs and neurons. Because some neurons did not show spontaneous activity during each period, the neuron number differs.

We next employed the marathon-experiments toward the crucial question of whether SV refilling during such harsh stimulus conditions is sufficient to keep the quantal size *q* at MNTB-LSO inputs constant. Several previous figures based on the scenario that this is the case (e.g., Figure 8I*a*). We analyzed sIPSCs during all 60-s recovery periods and the preceding normalization period, i.e., during a total of 660 s per neuron, and determined the peak amplitude. The analysis covered mIPSCs as well as multiple release events, including action potential-triggered ones. For Ctrls, the number of events amounted to 3,444 (*n* = 17 neurons), and the values for ALX and KO cohorts were 3,030 (*n* = 15) and 411 (*n* = 8), respectively. Ctrls displayed median values in the range of 20.5 – 25.6 pA (Figures 10A*a*,**B***a*,**C***a*) and no time-dependent trend, implying that *q* stayed constant over time. ALX sIPSC sample medians ranged from 17.7 to 18.8 pA, also displaying no trend toward changing *q* (Figures 10A*b*,**B***b*,**C***b*). Finally, KO medians ranged from 15.8 – 19.8 pA, and even here, *q* stayed constant (Figures 10A*c*,**B***c*,**C***c*). The values were also quite similar to the *q* values obtained above from Gaussian fits (cf. Table 3). Together, the results show that MNTB-LSO inputs are capable of refilling SVs reliably, even during prolonged stress conditions. It seems that MNTB axon terminals lacking functional GlyT2 possess a checkpoint mechanism that prevents the exocytosis of ‘empty' or minimally filled SVs (Liu, 2003). Metaphorically, they are thus able to avoid firing ‘blank cartridges' (for contrasting scenarios on vesicular transporters, see Cousin and Nicholls, 1997; Wojcik et al., 2004, 2006; Ruel et al., 2008). Our approach does not allow to record the exocytosis of empty SVs, because our recordings cannot assess presynaptic capacitance jumps. Therefore, we cannot exclude the extreme case of empty SV fusion with the plasma membrane. However, the release of partially filled SVs (>50%), and declining *q* amplitudes with time, would have been noticed via our approach.

### Modeling Reveals Substantial *RR* Deficiency During Acute GlyT2 Blockade Only If Inputs Are Very Heavily Stimulated

**Figure 11:**
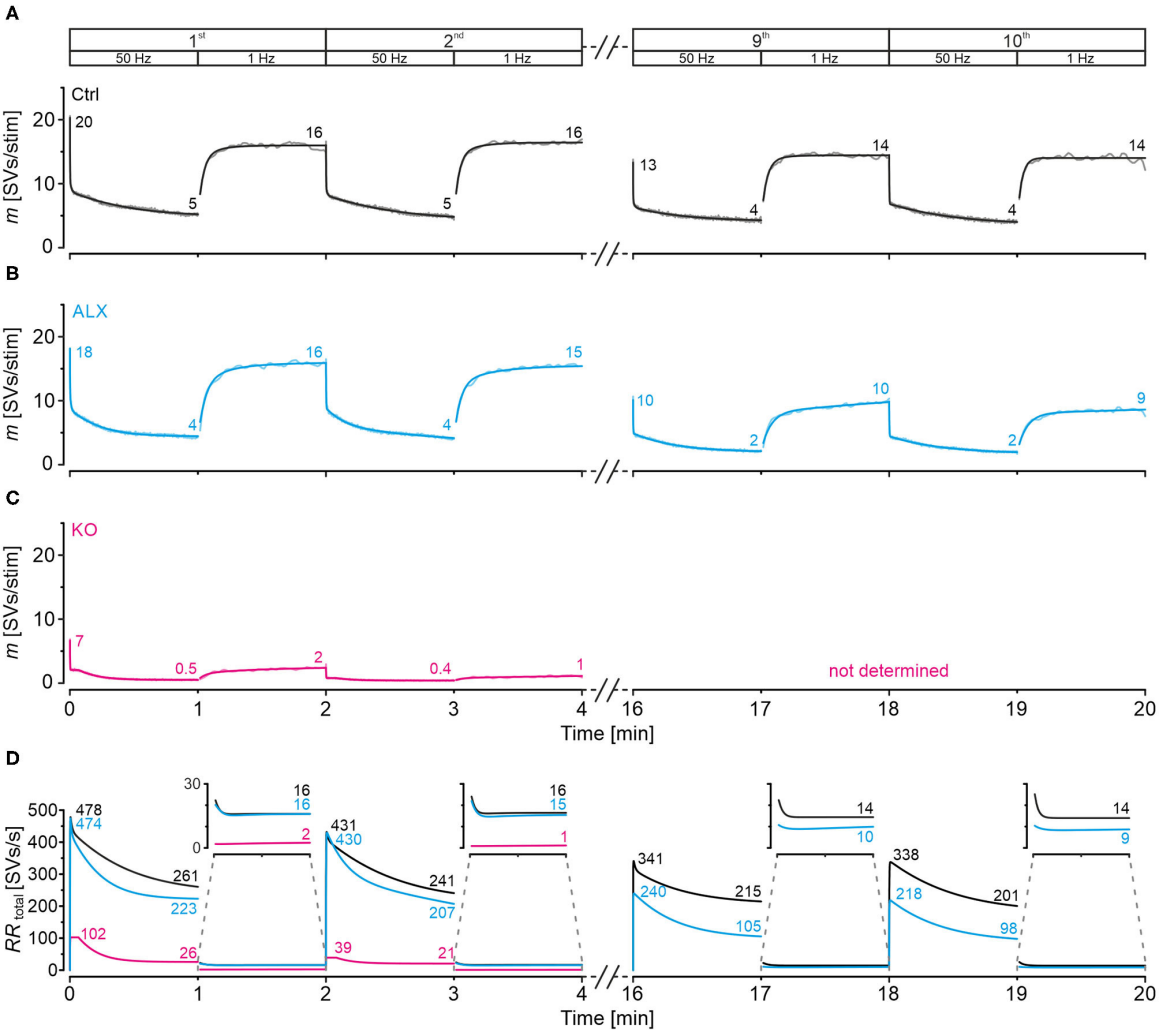
Docking site model points to moderately impaired replenishment when GlyT2 is acutely blocked and inputs are stimulated in marathon experiments. Chronic ablation of GlyT2 leads to severe replenishment deficiency. **(A–C)** Time course of *m* during challenge | recovery trains for Ctrl **(A)**, ALX **(B)**, and KO **(C)** samples (1st, 2nd, 9th, 10th train; in KOs only for 1st and 2nd train). Dark and light-shaded curves represent modeled data and empirical data, respectively. The model captures the empirical data very well. **(D)** SV replenishment kinetics (*RR*_total_) during challenge | recovery trains for Ctrl, KO, and ALX samples.

We also modeled SV release and replenishment behavior during marathon-experiments, again via SV-based computation. The model revealed that Ctrls manage to release 5 SVs/stim at the end of each 50-Hz train (Figure 11A). Moreover, Ctrls can reoccupy empty release sites very efficiently, as evidenced by a quantal content *m* of 16 SVs/stim after each 1-Hz recovery period. Their high-fidelity performance is achieved by high *RR*_*total*_ values during challenge and recovery periods (Figure 11D). Notably, the gradual decline during the 20-min experiment implies some fatigability in the long range (Figure 9A*a*).

Upon acute pharmacological GlyT2 blockade, MNTB-LSO inputs perform increasingly less robustly in marathon-experiments than Ctrls, as evidenced by gradually decreasing *m* values during both challenge and recovery periods (Figure 11B). During the final trains, values are ~1/2 and ~2/3 of the initial ones (2 vs. 4 SVs/stim for challenge; 9–10 vs. 14 for recovery). Likewise, *RR*_total_ is about 50% lower than in Ctrls during the end of challenge periods, particularly toward the end of the marathon-experiment (Figure 11D; 105 vs. 215 SVs/s in 9th train, 98 vs. 201 in 10th). Impaired reoccupation of empty sites is present during recovery periods of late trains [Figure 11D; 10 vs. 14 SVs/s in 9th train (71%), 9 vs. 14 in 10th (64%)]. Importantly, the differences are absent in early trains [16 vs. 16 SVs/s in 1st (100%), 15 vs. 16 SVs/s in 2nd (94%)].

GlyT2 gene deletion renders the few remaining MNTB-LSO inputs incable of performing continually (Figure 11C). At the end of the 1st recovery period, RR_total_ is low as 2 SVs/s, and only one empty site became replenished per second at the end of the 2nd recovery period (Figure 11D). Because of the heavy depression and virtually total collapse of neurotransmission during the first two trains (Figures 9C*a*, **11**C), we refrained from analyzing later trains in KOs.

### Manifold Replenishment of the Initial *RRP* Is Indefatigable at Ctrl MNTB-LSO Inputs, Moderately Affected Upon Pharmacological GlyT2 Blockade, and Severely Impaired in KOs

**Figure 12:**
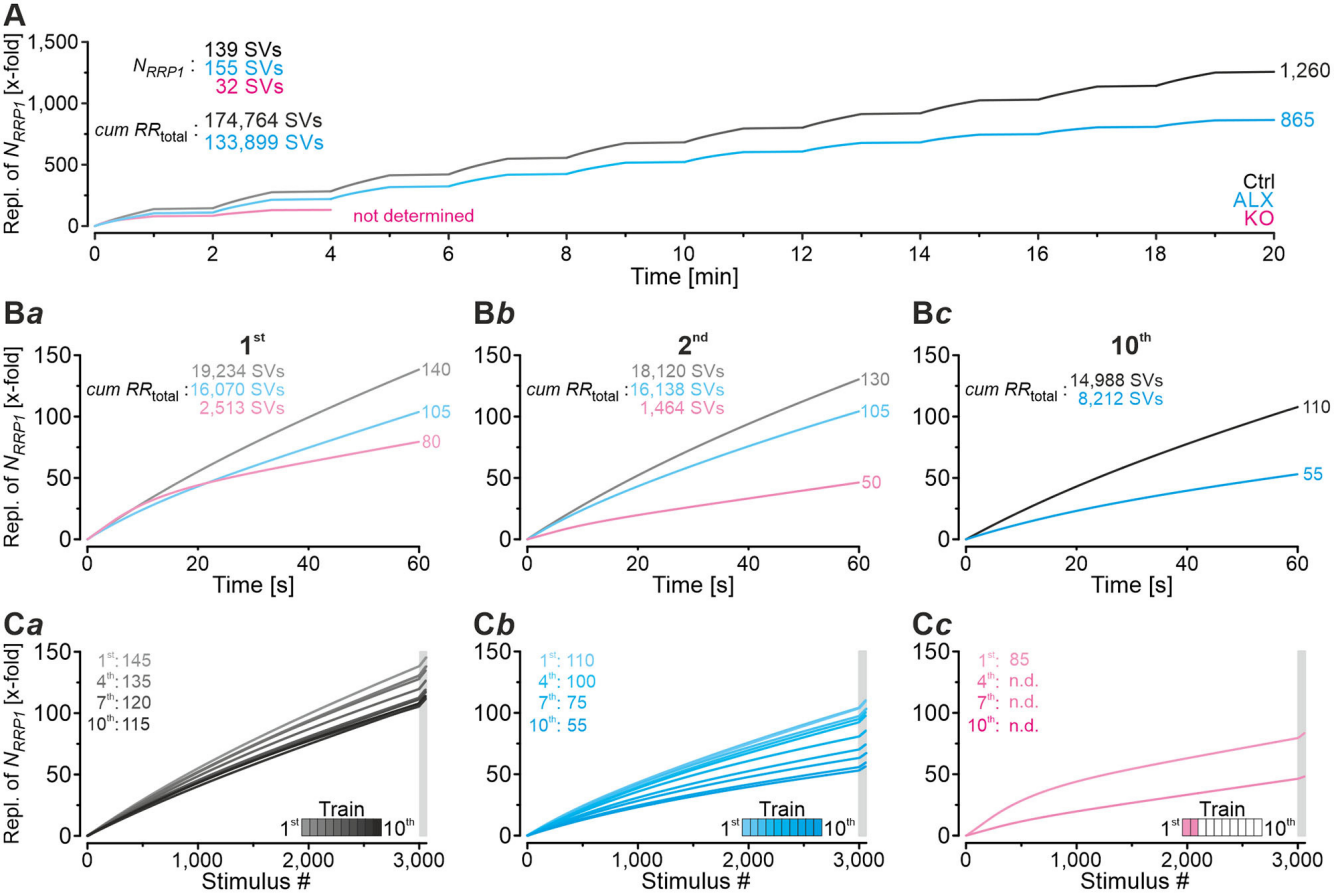
Comparison of manifold replenishment of *N*_*RRP*_. **(A)** Time course of x-fold replenishment of the initial *N*_*RRP*_ (*N*_*RRP*1_) for Ctrl, ALX, and KO samples (in KOs only for 1st and 2nd challenge | recovery trains). **(B)** Time expanded close-ups of *N*_*RRP*1_ replenishment during 1st **(B***a***)**, 2nd **(B***b***)**, and 10th challenge train **(B***c***)**. **(C)** *N*_*RRP*1_ replenishment during all 10 challenge | recovery trains plotted as a function of stimulus # for Ctrl **(C***a***)**, ALX **(C***b***)**, and KO **(C***c***)**. Gray boxes mark recovery periods. Trains are color-coded from light to dark.

In order to guarantee reliable synaptic transmission, *RRP*s need to be replenished many times and continually during prolonged stimulation. We wondered about the kinetics and extent of manifold replenishment of *RRP*_1_ at MNTB-LSO inputs and the differences caused by lacking GlyT2 re-uptake activity. To address the point, we further exploited the modeled data from the marathon-experiments in Ctrl, ALX, and KO recordings (for the latter, only trains 1 and 2) and plotted the number of SVs replenished during the 10 challenge/recovery trains in a cumulative manner (Figure 12). At the end of the marathon-experiments, replenishment added up to 174,800 SVs in Ctrls and 133,900 SVs in ALX (Figure 12A; data rounded to 100). As *N*_*RRP*1_ comprised 139 and 155 SVs, respectively, this calculated to a 1,260-fold turnover in Ctrls and a 865-fold turnover in ALX when 30,600 stimuli are presented (Figure 12A; data rounded to 5). Numbers during the 1st challenge period were 19,200, 16,100, and 2,500 SVs (Ctrl, ALX, KO), which related to 140-, 105-, and 80-fold replenishment of the respective *N*_*RRP*1_ (Figure 12B*a*). Corresponding numbers for the 2^nd^ challenge period were 18,100, 16,100, and 1,500 SVs (130-, 105-, 50-fold; Figure 12B*b*). During the 10th challenge period (minute 18–19), 15,000 and 8,200 SVs became replenished in Ctrls and ALX, respectively. corresponding to 110- and 55-fold turnover of *N*_*RRP*1_ (Figure 12B*c*).

The above results emphasize several aspects. (1) With functional GlyT2, *N*_*RRP*__1_ is replenished 35 times more often than upon pharmacological blockade of the transporter (3,000 stimuli at 50 Hz in 1st train). In other words, acute pharmacological blockade of GlyT2 decreases SV replenishment to 75% (105-fold/140-fold; Figure 12B*a*). The corresponding results for the 2nd challenge train are 81% (105/130; Figure 12B*b*), and for the 10th challenge train 50% (55/110; Figure 12B*c*). Integration throughout the marathon-experiment (till end of 10th train) yields ~70% (865/1,260) of the Ctrl value (Figure 12A). Consequently, GlyT2 activity appears to contribute ~20–30% of the glycine supply to the presynaptic MNTB axon terminals, and the effect increases with stimulus duration. (2) KOs display severely impaired SV replenishment during all trains, yet surprisingly they still manage to replenish *N*_*RRP*__1_ many times. During the 1st challenge period, replenishment is 80-fold, which calculates to 57% of the Ctrl value (80/140; Figure 12B*a*). Impaired SV replenishment in KOs becomes massive during the 2nd challenge period (38%, 50/130). Again, these results identify a prominent stimulus-time effect. Furthermore, the fact that KO inputs still manage to replenish *N*_*RRP*__1_ multifold points to a relatively large recycling pool that can be refilled in the absence of GlyT2. Consequently, there must be another source, or even several sources, for the supply of glycine to the vesicular transporter VIAAT, in addition to GlyT2 activity. (3) There are remarkable differences between ALX and KOs. In KOs, replenishment activity during challenge periods 1 and 2 is much lower than in the presence of ALX (1st: 76%, 80/105; 2nd: 48%, 50/105). These differences are unexpected and indicate that chronic elimination of GlyT2 impairs the replenishment machinery at various levels. Apparently, not only is GlyT2 re-uptake activity heavily reduced in KOs, but other glycine sources seem to be affected as well. (4) There are time effects within each cohort, but the extent varies considerably. In Ctrls, replenishment capacity declines to ~80% from the 1st to the 10th challenge train (110/140; Figures 12B*a*,*c*). Compared to the ALX cohort, which declines to ~50% (55/105), the reduction is moderate. We conclude from these observations high robustness and little fatiguability of SV replenishment at MNTB-LSO inputs with functional GlyT2, even under ongoing high-frequency stimulus conditions. Remarkably, GlyT2 transport contributes to these features particulary during later aspects of sustained stimulation (after minutes).

Time-dependent decline of replenishment behavior is most profound in KOs. Between the end of the 1st and 2nd challenge period, i.e., within 2 min and in response to 3,060 stimuli, replenishment capacity declines to ~60% (50/80; Figures 12B*a*,*b*). Ctrl inputs never decline to this low level, as they maximally fall to ~80% (110/140). For ALX, the ~60% level is not reached before the 8th train (65/105). When focusing at recovery periods alone, replenishment is apparently 5-fold in each cohort. It calculates to 145-fold – 140-fold for the 1st train in Ctrls (Figures 12B*a*,**C***a*), 110-fold−105-fold in ALX (Figures 12B*a*,**C***b*), and 85-fold – 80-fold in KOs (Figures 12B*a*,**C***c*). However, the impression is erroneous and due to rounding artifacts. Without rounding, replenishment is 7-, 6-, and 4-fold, respectively (145-138, 110-104, 83-79), confirming the different replenishment efficacy between groups, even during recovery periods with very little stimulation activity.

### Besides GlyT2, Asc-1 Is a Likely Glycine Source at MNTB-LSO Inputs

**Figure 13:**
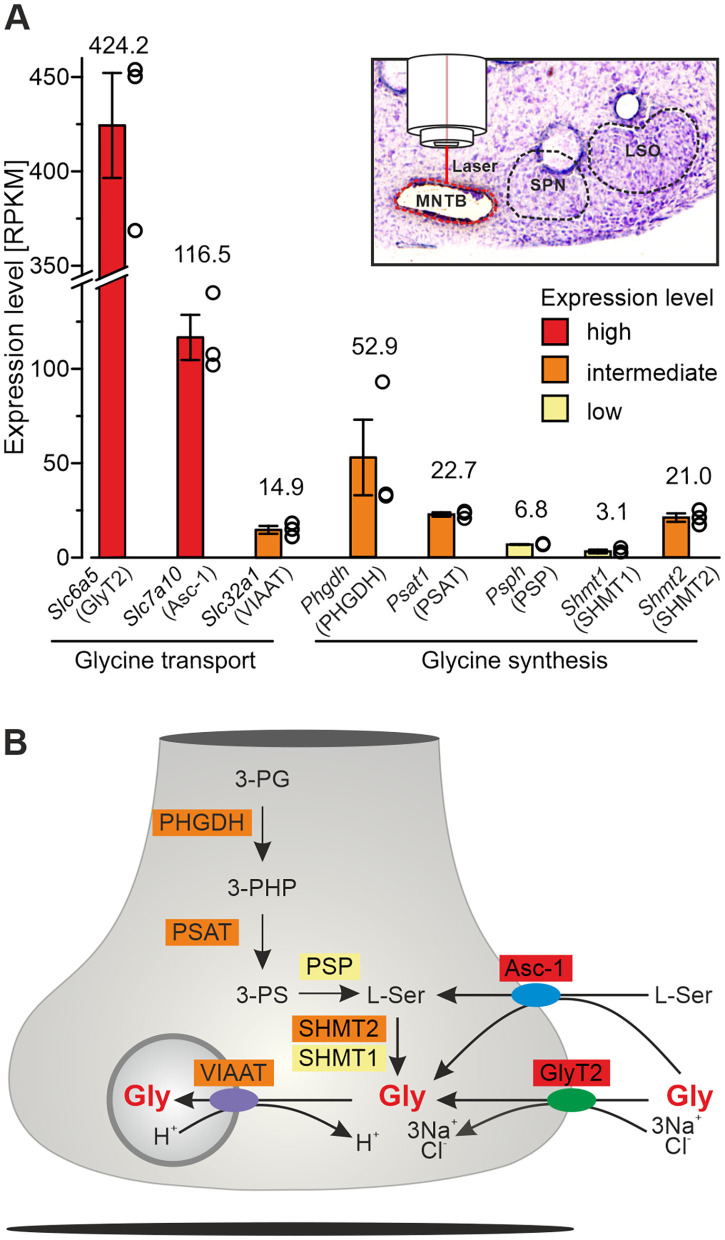
Gene transcripts corresponding to proteins involved in glycine synthesis and glycine transport are highly abundant in the MNTB. **(A)** Expression levels of different transcripts (sample data; respective protein symbols are shown in brackets). Inset depicts a Nissl-stained coronal slice from a P11 mouse with the SOC after laser microdissection of the MNTB. **(B)** Pathways for glycine synthesis and transport. Color code highlights high (red), intermediate (orange), and low (yellow) expression levels. Abbreviations: Asc-1, Asc-type amino acid transporter 1; Gly, glycine; L-Ser, L-serine; PG, phosphoglycerate; PHGDH, phosphoglycerate dehydrogenase; PHP, phosphohydroxypyruvate; PS, phosphoserine; PSAT, phosphoserine aminotransferase; PSP, phosphoserine phosphatase; SHMT, serine hydroxy methyltransferase. Notice that GlyT2- and Asc1-mediated uptake is directly coupled to glycine filling of SVs in the recycling pool.

Our results of ongoing glycinergic transmission, despite GlyT2 inactivation (Figures 9, 11, 12), point to other glycine sources for supply (Safory et al., 2015; Zafra et al., 2017; Mesuret et al., 2018). These sources might be other glycine uptake systems or proteins involved in glycine synthesis. We addressed this point by analyzing transcripts for such candidate proteins. Analysis involved sequencing the mRNA of laser-dissected MNTB tissue obtained from postnatal day 11 slices (Fischer et al., 2019; Müller et al., 2019). The most highly abundant transcripts were *Slc6a5* and *Slc7a10*, which encode the proteins GlyT2 and Asc-1, respectively (Figure 13A). Expression levels of several genes coding for proteins involved in glycine synthesis were intermediate. Together, the transcriptome results reveal Asc-1 as a very good candidate for glycine re-uptake at MNTB axons in cooperation with GlyT2 and suggest that glycine synthesis as another source for glycine supply (Figure 13B). Immunohistochemical analyses of Asc-1 have been done in mice and rats, and prominent Asc-1 immunoreactivity was reported for the superior olivary complex, without mentioning individual nuclei (Helboe et al., 2003). Recently, it was reported that Asc-1 is enriched in the mouse brainstem and spinal cord, and that the distribution correlates with regions of high-density glycinergic activity (Ehmsen et al., 2016). Asc-1 facilitates the transport of several small neutral amino acids (including glycine, L-serine, L-threonine, L-alanine, and L-cysteine) in a Na^+^-independent manner and operates preferentially in an exchange mode (Fukasawa et al., 2000; K_*m*_ values: 7.8, 11.3, 19.3, 23.0, and 23.7 μM, respectively). In line with this, the phenotype of *Slc7a10* KO mice (tremors, ataxia, rigidity, seizure-like events) reflects impaired glycinergic inhibitory transmission (Safory et al., 2015).

## Discussion

**Figure 14:**
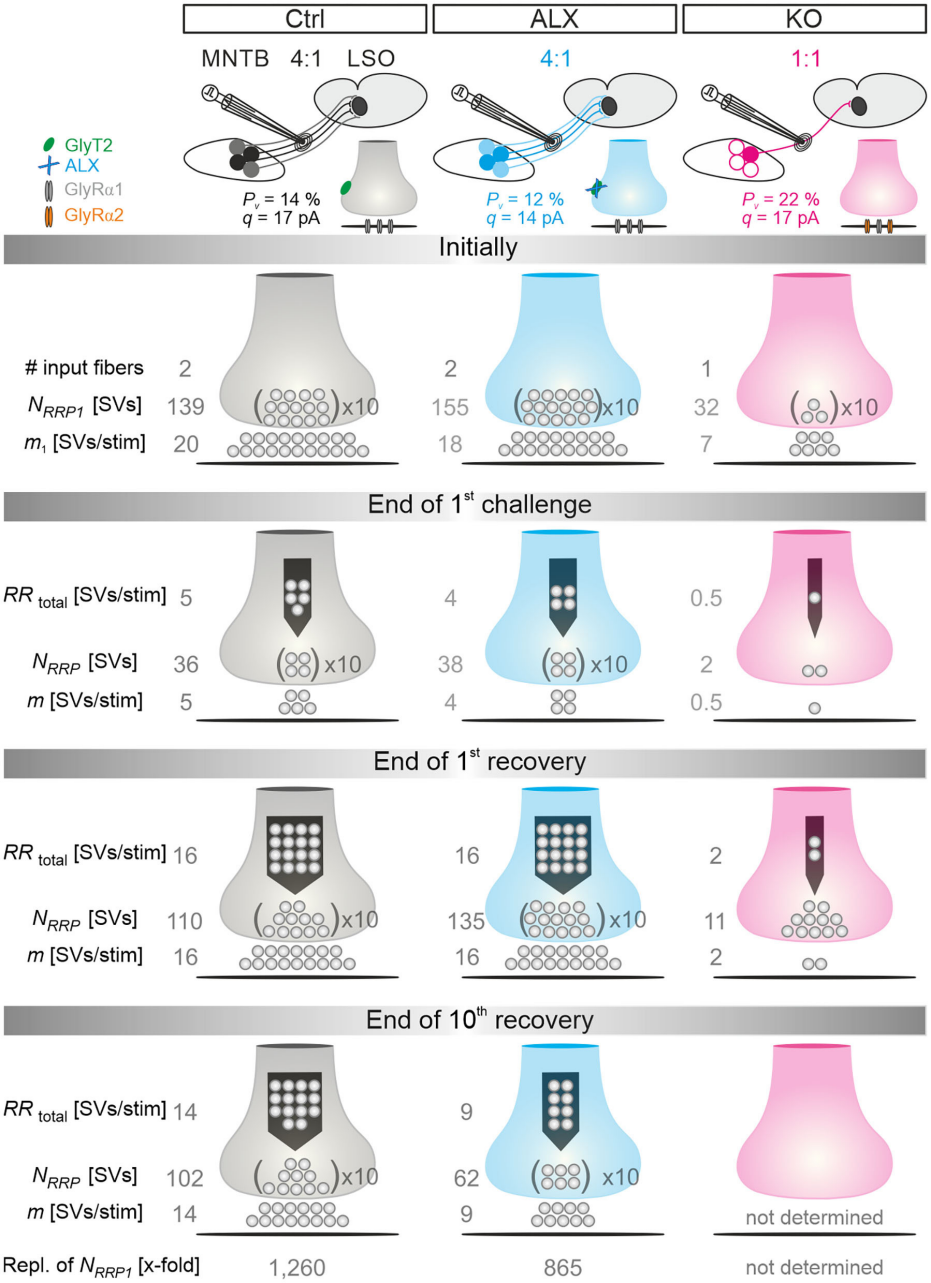
Graphical summary of the main results of the present study. Schematic views comparing the functional organization of MNTB-LSO inputs between Ctrl, ALX, and KO groups. In the presence of functional GlyT2 (Ctrl), ~4 presynaptic MNTB neurons form functional synapses with a single postsynaptic LSO principal cell in P11 mice (4:1 convergence). Inputs maintain robust neurotransmission even during prolonged high-frequency stimulation lasting 20 min and involving 30,600 stimuli (10 trains; 50 Hz | 60 s challenge, 1 Hz | 60 s recovery), and they manage to replenish their initial *N*_*RRP*_ 1,250-fold. Upon genetic ablation of GlyT2 (KO), the input number is reduced to ~1 (1:1 convergence), implying massive functional degeneration of the microcircuit and an important role of GlyT2 during circuit refinement. This conclusion is corroborated by results that point to a missing developmental shift in the subunit composition of the postsynaptic glycine receptors (GlyR). In line with the reduced input number goes a reduced input strength and a ~4-fold smaller *N*_*RRP*_. KO synapses display a 50% higher release probability *P*_*v*_ (22 vs. 14%), indicating ‘attempts' to compensate for the reduced *N*_*RRP*_. Upon sustained high-frequency stimulation (half-maximal amplitude, thus only 2 input fibers are activated in Ctrls and ALX, cf. light- and dark-shaded somata in top panels), SV replenishment is severely impaired in GlyT2 KOs, resulting in drastically smaller *N*_*RRP*_ values. However, neurotransmission does not collapse completely, implying a depot of glycine-filled SVs that is independent of GlyT2 activity. The finding that the quantal size *q* does not differ from Ctrls (17 pA in both cases) provides further support to this conclusion. Upon acute pharmacological blockade of GlyT2 (ALX), MNTB-LSO inputs still perform robustly, but they are less fatigue-resistant to high-frequency challenge than Ctrl inputs. The quantal size remains unchanged. When exposed to 30,600 stimuli in 20 min, inputs manage to replenish the initial *N*_*RRP*_ 865-fold as compared to 1,250-fold in Ctrls. The surprisingly small difference of ~30% can be attributed to GlyT2 activity, and the results implicate that glycine reuptake by GlyT2 is not the most important source for refilling the recycling pool with glycine.

The present paper provides the first view of the role of a neurotransmitter re-uptake system during sustained high-frequency neurotransmission. We have analyzed MNTB-LSO inputs, a fast and robust glycinergic connection in the auditory brainstem. GlyT2 activity was abolished genetically and via acute pharmacological blockade, and prolonged stimulation, comprising up to 30,600 stimuli in 20 min, was used to heavily challenge the inputs. Four key findings emerge from the study (Figure 14): (1) Whereas ~4 MNTB neurons converge on a single LSO neuron in Ctrls, the number is drastically reduced to ~1 in GlyT2 KO mice. Thus, GlyT2 appears to contribute to synapse maturation and maintenance. Because these findings about the input ratio are based on electrophysiology, morphological evidence is required to corroborate this point via an independent approach. (2) Longer decay times of IPSCs in GlyT2 KOs, together with results from pentobarbital experiments, point to the possibility that the normally occurring developmental shift from “fetal” α_2_ to “adult” α_1_ subunits does not take place at LSO neurons upon gene ablation. Further histological and pharmacological analyses are needed to corroborate this hypothesis. (3) Computational modeling shows that Ctrl MNTB-LSO inputs manage to replenish *N*_*RRP*_ 140-fold when stimulated at 50 Hz | 60 s. Under acute pharmacological blockade, replenishment is 105-fold, and it is still 80-fold in KOs. Therefore, replenishment is not completely abolished in KOs and only moderately affected upon pharmacological GlyT2 blockade. (4) When GlyT2 is acutely inactivated and inputs are heavily challenged with 30,600 stimuli, replenishment is ~30% lower than in Ctrls (865 vs. 1,260-fold of *N*_*RRP*1_). The ongoing glycinergic transmission, despite inactive GlyT2, points to a 2nd source for glycine supply, if not several sources. Transcriptomics data show that the amino acid transporter Asc-1 may be such a source. We conclude that glycine re-uptake by GlyT2 is an important, yet not the most crucial factor in the SV recycling pathway rendering MNTB-LSO inputs resilient to synaptic depletion.

### Multifold and Indefatigable *RRP* Replenishment Guarantees Robustness of MNTB-LSO Inputs

The results from our marathon-experiments demonstrate that postnatal day 11 MNTB-LSO inputs withstand strong stimulation conditions impressively well (Figures 9, 11, 12). The inputs can do so because they possess a very efficient replenishment machinery. Via computational modeling, we estimated a 140-fold replenishment of *N*_*RRP*1_ during the 1st 50 Hz | 60 s train, calculating to an average *RR*_*total*_ of 320 SVs/s (19,234 SVs in 60 s), 2.3 times/s (140 times/60 s), or ~430 ms (1,000 ms/2.3) to refill an empty release site (Figures 11D, **12B***a*). Notably, maximal *RR*_*total*_ is 478 SVs/s (Figure 11D). The above values do not change considerably during the 2nd challenge train (130-fold [Figure 12B*b*], 300 SVs/s, 2.2 times/s, ~450 ms to reoccupy an empty site). Even during the 10th challenge train, replenishment is still strong (110-fold [Figure 12B*c*], 250 SVs/s, 1.8 times/s, ~560 ms to reoccupy an empty site), although the replenishment rate [SVs/s] declines to 80% and the time required to reoccupy an empty site increases to 130% compared to the 1st train. Therefore, MNTB-LSO inputs are able to resupply filled SVs to the *RRP* in the presence of GlyT2 transport activity, even under sustained high-frequency stimulation conditions.

To our knowledge, above *RRP*-based calculations have not been made before, and we are unaware of any study that has addressed sustained synaptic performance and *RRP* replenishment to an extent as we did here (20 min). In one study that comes close, cerebellar mossy fiber-granule cell inputs were analyzed in two 100 Hz | 60 s trains interrupted by a 10-s recovery interval (Saviane and Silver, 2006). The releasable pool was refilled at 8 SVs/s. We report averages of 302 and 250 SVs/s for the 1st and 10th train, respectively. At a glutamatergic calyx of Held-MNTB input, ~20,000 SVs are released within 4 s upon 50-Hz stimulation (Grande and Wang, 2011; Hallermann, 2015). As the N_*RRP*_ of this giant synapse comprises ~1,500 SVs (Neher and Sakaba, 2008; Neher, 2010), this corresponds to a 13-fold release of the *RRP* during this period, equivalent to ~3-fold/s. Our Ctrl recordings show that MNTB-LSO inputs release 1,800 SVs during the first 4 s of a 50-Hz train, which also corresponds to a 13-fold turnover. Consequently, and possibly not by chance, the release rate is virtually identical at these sequentially aligned inputs in the auditory pathway (calyx of Held-MNTB, then MNTB-LSO synapses). The *N*_*RRP*_ of 139 SVs in the Ctrl cohort is distributed among ~2 MNTB input neurons that converge on a single LSO neuron (Figures 11A, **13**). This relates to ~70 SVs per input neuron. Together with the *RRP*, a recycling pool and a resting pool form the total pool of SVs (nomenclature after Alabi and Tsien, 2012). Replenishment of the *RRP* is achieved via the recycling pool which in turn is supplied by the resting pool. The total number of SVs, i.e., the sum across all pools, is unknown for MNTB-LSO axon boutons (Cant, 1984; Helfert and Schwartz, 1986; Helfert et al., 1992; Brunso-Bechtold et al., 1994; Gjoni et al., 2018a) and requires ultrastructural 3D analysis via electron microscopy, particularly in mice. Data from other systems indicate that *RRP*s amount to 0.15–5% of the total number of SVs (Südhof, 2000; Rizzoli and Betz, 2005; Nouvian et al., 2006; Gan and Watanabe, 2018). Assuming that the *RRP* contributes 1% to the total pool size of MNTB-LSO inputs, the total pool would comprise ~14,000 SVs. This raises the question of how long MNTB-LSO inputs can feed on this total pool depot without the need to refill endocytosed SVs with glycine. Our modeling results show that 14,000 SVs are released within ~40 s during the 1st train (Figure 12B*a*). The 40-s value is in accordance with literature data showing that replenishment of the recycling pool via recruitment of refilled SVs starts after tens of seconds (de Lange et al., 2003; Rizzoli, 2014; Watanabe et al., 2014). Interestingly, MNTB-LSO inputs are capable of responding reliably (with little failures, Figure 4E*a*) to sustained 50-Hz stimulation before the total pool of SVs is used up and they get under considerable pressure to replenish. Together, the data indicate that MNTB-LSO inputs possess a large total pool to feed on. It will be interesting to analyze whether other synapse types differ in this respect.

### Synaptic Performance Upon Acute Pharmacological Blockade of GlyT2

The findings with acute pharmacological GlyT2 inactivation by ALX vary only to a minor degree from the Ctrl results (e.g., Figure 8). Modeling determined a 105-fold replenishment of *N*_*RRP*1_ during the 1st challenge train, corresponding to mean values of ~1.8 times/s, ~270 SVs/s (maximal *RR*: 474 SVs/s, Figure 11D), and ~560 ms to reoccupy an empty site (Figures 11D, 12B*a*). Very similar values are obtained for the 2nd train (Figure 12B*b*). However, by the 10th train, values have changed considerably (55-fold [Figure 12B*c*], 0.9 times/s, ~140 SVs/s, ~1.1 s to reoccupy an empty site). Concomitant with the changes, *RR* declines to ~50% between the 1st and the 10th train, and the time required to reoccupy an empty site increases to ~130%. In essence, these data demonstrate the vulnerability of MNTB-LSO synapses when glycine supply via GlyT2 is impaired. Whereas, the replenishment rate at Ctrl inputs declines to 80%, the decline goes down to 50% when GlyT2 activity is pharmacologically blocked. Furthermore, the total pool of SVs, assumed to comprise 15,500 SVs, is used up after ~57 s, yet eIPSC amplitudes are still >25% of the baseline (Figures 9B*a*, **12B***a*). Together, these results corroborate the conclusion that synaptic transmission and *RRP* replenishment do not crucially depend on GlyT2 re-uptake activity. The dependence on GlyT2 becomes obvious only during the 2nd half of marathon-experiments (Figures 9B, 11D).

Effects of pharmacological GlyT2 inactivation on synaptic transmission have been investigated quite extensively in the spinal cord, but only a few studies have analyzed eIPSCs. The rate, amplitude, and decay time of sIPSCs in the presence of ALX (200 nM) were statistically indistinguishable from Ctrl recordings (Eckle and Antkowiak, 2013). In contrast, ALX treatment was reported to decrease and prolong mIPSCs (Jeong et al., 2010). Pharmacological inhibition of GlyT2 with ORG25543, an irreversible GlyT2 inhibitor (Morita et al., 2008; Mingorance-Le Meur et al., 2013), increased the decay time of glycinergic mIPSCs, sIPSCs, and eIPSCs, and inhibition for 15–24 h resulted in almost complete loss of inhibition (Bradaïa et al., 2004; Rousseau et al., 2008). eIPSCs were also analyzed in the Jeong paper, and like the mIPSCs, they became smaller in amplitude and lasted longer upon ALX treatment. The increased decay times of eIPSCs are in line with our findings, but we did not observe decreased eIPSC amplitudes (cf. Figures 7D*a*,*b*). The Rousseau paper applied sustained stimulation for 30-60 min, but only at a frequency of 1 Hz and lower. Our study is the first which assessed synaptic transmission under long-lasting stimulation conditions (10 × 50 Hz | 60 s).

### Synaptic Performance Upon Genetic Ablation of GlyT2

KO MNTB-LSO inputs are not completely silenced, but they display several severe deficits. Their *N*_*RRP*1_ comprises only 32 SVs located in a single MNTB input neuron (1:1 convergence), which implies massively degenerated functional connectivity at the cellular level. In comparison, Ctrl inputs displayed 139 SVs in ~2 MNTB neurons (~70 SVs/neuron), and 4 MNTB input neurons converge on a given LSO principal neuron. During the whole 60 s of the 1st 50-Hz train, KO inputs replenish the *RRP* 80-fold (Figure 12B*a*; 1.3 times/s, 42 SVs/s, 750 ms to reoccupy an empty site; maximal *RR*: 102 SVs/s, Figure 11D). Interestingly, during the first 10 s of the 1st train, replenishment of *RRP*_1_ looks very similar to that of Ctrl and ALX synapses (Figure 12B*a*). Indeed, KO inputs appear to possess an SV pool that enables them to release ~900 SVs and, therefore, to replenish *RRP*_1_ ~30-fold during these first 10 s (corresponding to 3 times/s), despite the loss of GlyT2. Between second #10 and the end of the 1st train, however, *RRP* replenishment at KO inputs is only 50-fold, corresponding to once per second (Figure 12B*a*). In contrast, the replenishment capacity barely changes at Ctrl and ALX inputs throughout the 1st train. During the 2nd train, replenishment at KO inputs declines further (0.83 times/s, 24 SVs/s, ~1.2 s to reoccupy an empty site; Figures 12B*b*,**C***c*). Consequently, *RR* declines to ~55% of the value from the 1st train ([24 SVs/s]/[43 SVs/s]). In comparison, ALX synapses decline to a 55% value between the 1st and the 10th train, and Ctrl synapses never decline below 80%, demonstrating their resilience. We were unable to assess the synaptic performance of KO inputs during the 3rd and subsequent trains, because most eIPSCs sank below the threshold level (Figure 9C).

Altogether, KO inputs release ~2,500 SVs during the 1st train (Figure 12B*a*), of which only 0.5 SVs/stim are released at the train's end (Figure 11C). Assuming that the total pool contains 3,200 SVs (*N*_*RRP*1_ = 32 SVs), ~700 SVs would be left, a depot large enough to be used. However, the vast majority of responses are failures after ~10 s (Figures 4E*a,b*), suggesting an almost totally depleted *RRP*. We therefore conclude that KO inputs not only possess a smaller *RRP* than Ctrls, but they also have smaller recycling and resting pools. Consequently, the total depot size appears to be smaller than 3,200 SVs and it likely comprises 2,000–2,500 SVs.

To our knowledge, no previous study has assessed synaptic performance upon inactivation of a presumptive key molecule, in this case GlyT2, during prolonged high-frequency stimulation (>30,000 stimuli). In the original study of GlyT2 KO mice (Gomeza et al., 2003b) and a follow-up paper (Latal et al., 2010), the authors analyzed sIPSCs and action potential-independent mIPSCs in hypoglossal motoneurons. In contrast to our findings (cf. Figure 2D*b*), mIPSC amplitudes (representing *q*) and sIPSC amplitudes were reduced to about half, whereas the decay time was unchanged (cf. Figure 2D*c*). Regarding a lower release rate, our results comply with those from the Gomeza and Latal papers (cf. Figure 2D*a*). Radioactive uptake assays revealed a residual glycine transport of ~30% in the hypoglossal nucleus and the spinal cord of GlyT2 KOs (Gomeza et al., 2003b), lower than implied by our results. Collectively, the findings reveal more differences than similarities between the two glycinergic synapse types (hypoglossal nucleus and MNTB-LSO) and point to synapse heterogeneity. Remarkably, no study has reported a complete loss of glycinergic transmission in GlyT2 KOs, providing further evidence that GlyT2 is not the only supplier of glycine at synapses using this transmitter. Our transcriptomics data indicate that Asc-1 is an additional glycine transporter candidate and that the glycine synthesis pathway may also be of relevance (Figure 13). In this context, it is interesting that GlyT2 KO mice, despite severely impaired glycinergic inhibition, can still produce sufficient expiratory airflow to produce ultrasound vocalization (Hülsmann et al., 2019).

### Genetic Ablation of GlyT2 Causes Drastically More Severe Deficits Than Acute Pharmacological Blockade, Implying Developmental Effects

Our study demonstrates a few similarities and several major differences between chronic and acute inactivation of GlyT2. A similarity is that neither chronic nor acute inactivation abolishes synaptic function completely, implying that GlyT2 is not the exclusive supplier of glycine for SV filling with this transmitter. A very prominent difference is the massively reduced synaptic strength in KOs, whereas the ALX cohort is normal in this respect. The reduction in KOs is associated with a loss of functional inputs and a smaller *RRP*, and the effects may involve synapse degeneration. Another difference is the longer IPSC decay time constant τ_*w*_ in KOs which we did not observe in ALX. We attribute this difference to a developmental role of GlyT2 in postsynaptic receptor maturation. Furthermore, the replenishment machinery of KOs is drastically impaired and leads to a fast collapse of transmission, whereas replenishment is only little affected in the ALX cohort. Synapses in the latter manage to maintain synaptic transmission during prolonged high-frequency challenge quite well and do not decline before 10 min. Taken together, the results imply chronic developmental impairments upon gene deletion that occur in addition to acute effects. The 1:1 convergence at KO MNTB-LSO inputs (Figures 3, 14) suggests that the death of GlyT2 KO mice during the 2nd postnatal week may be related to degeneration of synapses. We cannot say at present whether the degeneration is merely functional or also structural. To address this issue in further detail, morphological evidence is required. Chronic elimination of GlyT2 appears to impair the replenishment machinery at various levels. GlyT2 re-uptake activity is heavily reduced in KOs, and synaptic viability appears to be no longer maintained. Thus, our study adds a developmental aspect to GlyT2 function, namely a role in maturation and maintenance of functional glycinergic synapses and microcircuits, as suggested earlier (Friauf et al., 1999).

### Readily-Releasable SVs per Active Zone

In maximal stimulation experiments, we determined that ~4 MNTB fibers converge on a single LSO principal neuron in Ctrls, confirming results from previous functional studies (Kim and Kandler, 2003; Walcher et al., 2011; Gjoni et al., 2018b; Müller et al., 2019). However, we performed the vast majority of challenge | recovery experiments at half-maximal stimulus amplitudes in which we activated most likely ~2 MNTB fibers. Future analysis should employ minimal stimulation experiments to assess single fiber properties (e.g., strength, quantal content, performance during sustained high-frequency stimulation) and compare them between both genotypes.

Recent ultrastructural analysis of axons found a prevalence of large inhibitory axons on the soma and proximal dendrites of LSO neurons, corroborating the powerful glycinergic inhibition of these cells (Gjoni et al., 2018a). Per axon, an average of six varicosities (terminal boutons and *en passant* swellings) were reported, with a mean of 2.5 presynaptic active zones (AZs, i.e., transmitter release zones of 0.2–0.5 μm diameter; Südhof, 2012) per varicosity. Thus, we estimate that the ~2 MNTB units activated in the present study with half-maximal stimulus amplitudes display ~30 AZs. Considering an *N*_*RRP*_ of ~140 SVs, we estimate ~5 release-competent SVs per single AZ. At the calyx of Held, there are 2-3 docked SVs/AZ (Rollenhagen and Lübke, 2006; if the *RRP* is defined by ~2 SV diameters [60 nm], the value is ~18 SVs/AZ; Rollenhagen and Lübke, 2010). The same paper reports 40 SVs/AZ at mossy fiber boutons in the hippocampus. Finally, 7-8 docked SVs are found at individual terminals of climbing and parallel fibers (Xu-Friedman and Regehr, 2004). In conclusion, MNTB-LSO inputs are not exceptional regarding the number of release-competent SVs at a single AZ. Since Ctrls release ~4,000 SVs during 10 s of 50-Hz stimulation (Figure 12B*a*), 133 SVs appear to be exocytosed from each AZ, relating to 13 SVs/s. The above estimates and calculations are speculative, and the actual number of fusion-competent SVs per AZ and MNTB varicosity needs to be determined by 3D electron tomography (Imig et al., 2014).

### Technical Aspects of the Computational Model

The computational model applied in the present study to calculate *RR* is based on modeling the dynamics of a single vesicular pool. It quantifies the *RRP* size as a function of time, where *RR* is phenomenologically modeled to decay exponentially with two time constants (equation 3). This approach contrasts our earlier work (Brill et al., 2019), in which the decay was modeled with a Hill equation. In the present model, we assume that the initial *RRP* size is maintained throughout the experiment, i.e., the number of release sites available for replenishment remains constant and equals *N*_*RRP*1_. We also assume that *RR*_*total*_ is proportional to the number of empty sites [cf. (Thanawala and Regehr, 2013; but see also Pulido and Marty, 2018)]. Evidence that the rate of vesicle replenishment varies linearly with *N*_*RRP*_ supports this assumption (Babai et al., 2010). If the pool is still partially filled, refilling is slower. Finally, we also assume *P*_*v*_ to stay constant, in contrast to a recently applied analytical model (Thoreson et al., 2016). Developing mechanistic multiple vesicle-pool models to explain long-term dynamics is a promising direction of future modeling work. In a separate paper, we will address this more.

## Concluding Remarks

High-frequency signaling at glycinergic auditory synapses is not restricted to brief bursts but can be sustained over 10 of s to min without accommodating. One outstanding feature is rapid and continuous replenishment of release-ready SVs. Genetic ablation of GlyT2 impairs replenishment but does not completely abolish synaptic transmission. From this we conclude that MNTB-LSO inputs possess further mechanisms, aside from GlyT2 activity, to refill their SV pools. Several open questions remain from our study. (1) Do MNTB neurons in GlyT2 KOs display altered biophysical properties, altered axonal arbors, and altered SV pools? (2) Can KO inputs recover fully, e.g., if recovery periods >60 s are introduced and no test stimuli are applied? (3) Do MNTB-LSO inputs behave differently to long-lasting high-frequency stimulation if all MNTB neurons converging on a given LSO are challenged with maximal stimulation amplitude? (4) Does GlyT2 gene deletion affect the molecules involved in orchestrating glycinergic neurotransmission at the MNTB-LSO inputs, such as the elimination of fetal α2GlyRs or the abundance and distribution of GlyT1? (5) Might different input modalities (aside from glycinergic, i.e., GABAergic) contribute to sIPSCs and explain blurred Gaussian distributions (cf. Figure 2B)? (6) Does Asc-1 act as an alternative glycine transporter and in cooperation with GlyT2? (7) Finally, it is interesting to use a combination of fluctuation analysis methods to examine quantal parameters more intensely during challenge trains (Silver, 2003; Saviane and Silver, 2006). We plan to address these issues in future studies.

## Data Availability Statement

The original contributions presented in the study are included in the article/supplementary materials, further inquiries can be directed to the corresponding author/s.

## Ethics Statement

Ethical review and approval were not required for this study, as the German law permits organ extraction after mice have been euthanized. Animal breeding and experiments were approved by the regional council according to the German animal protection act (TSchG § 4, Absatz 3) and in accordance with EU Directive 2010/63/EU.

## Author Contributions

EF, FK, and SB: initiation and concept of the project. SB, AM, CK, FK, and MF: acquisition of data. SB, AM, AS, and EF: analysis and interpretation. EF, SB, AM, and AS: writing of the manuscript. The authors had full access to all data in the study and take responsibility for the integrity of the data and accuracy of the analysis.

## Conflict of Interest

The authors declare that the research was conducted in the absence of any commercial or financial relationships that could be construed as a potential conflict of interest.

## Abbreviations

ACSF: artificial cerebrospinal fluid
ALX1393: O-[(2-Benzyloxyphenyl-3-flurophenyl)methyl]-L-serine
AZ: active zone
BSA: bovine serum albumine
Ctrl: control
CNQX: 6-cyano-7-nitroquinoxaline-2,3-dione
d: dorsal
DABCO: 1,4-diazabicyclo[2.2.2]octane
eIPSC: evoked IPSC
*f*: stimulus frequency
*FR*: fractional recovery
*g*: mixing constant
GABA: γ-aminobutyric acid
GABA_A_R: GABA_A_ receptor
GlyR: glycine receptor
GlyT1: glycine transporter 1
GlyT2: glycine transporter 2
IPSC: inhibitory postsynaptic current
*I*_*RRP*_: cumulative current amplitude upon complete depletion of *RRP*
k: number of comparisons
KO: GlyT2 knockout
l: lateral
LSO: lateral superior olive
L-Ser: L-serine
*m*: quantal content
*M*: number of release sites
mIPSC: miniature IPSC
MNTB: medial nucleus of the trapezoid body
n: number of recorded cells
n.d.: not determined
n.s.: not significant
*N*_*RRP*_: number of SVs in *RRP*
N_*RRP*1_: initial
*N*_*RRP*;_*P*: probability
PBS: phosphate-buffered saline
Pbt: pentobarbital
PFA: paraformaldehyde
PG: phosphoglycerate
PHGDH: phosphoglycerate dehydrogenase
PHP: phosphohydroxypyruvate
PS: phosphoserine
PSAT: phosphoserine aminotransferase
PSP: phosphoserine phosphatase
*P*_*v*_: release probability, probability of SV fusion upon an action potential
*q*: quantal size
RPKM: reads per kilobase per million
*RR*: replenishment rate
*RR*_*per*__emptysite_: *RR* per empty site
*RR*_min_: minimal *RR*
RR_recmin_: minimal *RR* during recovery
*RR*_recmax_: maximal *RR* during recovery
*RR*_total_: total *RR*
*RRP*: readily releasable pool
*RRP*_1_, initial *RRP*: before high-frequency challenge
SHMT: serine hydroxy methyltransferase
sIPSC: spontaneous IPSC
SPN: superior paraolivary nucleus
STD: short-term depression
SV: synaptic vesicle τ_*w*_ weighted decay time constant
U: Mann-Whitney U-test
VIAAT: vesicular inhibitory amino acid transporter.
